# 3-Arylidene-2-oxindoles as Potent NRH:Quinone Oxidoreductase 2 Inhibitors

**DOI:** 10.3390/molecules28031174

**Published:** 2023-01-25

**Authors:** Natalia A. Lozinskaya, Elena N. Bezsonova, Meriam Dubar, Daria D. Melekhina, Daniil R. Bazanov, Alexander S. Bunev, Olga B. Grigor’eva, Vladlen G. Klochkov, Elena V. Sokolova, Denis A. Babkov, Alexander A. Spasov, Sergey E. Sosonyuk

**Affiliations:** 1 Department of Chemistry, Lomonosov Moscow State University, 119991 Moscow, Russia; 2 Medicinal Chemistry Center, Togliatti State University, 445020 Togliatti, Russia; 3 Department of Pharmacology & Bioinformatics, Volgograd State Medical University, 400131 Volgograd, Russia

**Keywords:** oxindoles, indolin-2-ones, NQO2, NRH:quinone oxidoreductase 2, 3-arylidene-2-oxindoles, anticancer

## Abstract

The enzyme NRH:quinone oxidoreductase 2 (NQO2) plays an important role in the pathogenesis of various diseases such as neurodegenerative disorders, malaria, glaucoma, COVID-19 and cancer. NQO2 expression is known to be increased in some cancer cell lines. Since 3-arylidene-2-oxindoles are widely used in the design of new anticancer drugs, such as kinase inhibitors, it was interesting to study whether such structures have additional activity towards NQO2. Herein, we report the synthesis and study of 3-arylidene-2-oxindoles as novel NRH:quinone oxidoreductase inhibitors. It was demonstrated that oxindoles with 6-membered aryls in the arylidene moiety were obtained predominantly as *E*-isomers while for some 5-membered aryls, the *Z*-isomers prevailed. The most active compounds inhibited NQO2 with an IC_50_ of 0.368 µM. The presence of a double bond in the oxindoles was crucial for NQO2 inhibition activity. There was no correlation between NQO2 inhibition activity of the synthesized compounds and their cytotoxic effect on the A549 cell line.

## 1. Introduction

Human NRH:quinone oxidoreductase 2 (NQO2) is an enzyme that belongs to the quinone oxidoreductase gene family. The biological activity of NQO2 is quite multifaceted. NQO2 can be considered a detoxifying agent because it catalyzes two-electron reduction of quinones and quinoid compounds into hydroquinones [1]; however, NQO2 activity was also associated with ROS production [2,3,4]. This dual toxifying/detoxifying action of NQO2 is still being debated [5].

NQO2 was linked to the development of various diseases such as neurodegenerative disorders [3,6,7,8,9], malaria [10], glaucoma [11], COVID-19 [12] and various cancers. It is important to note that the role of NQO2 in cancer pathogenesis is still being discussed. It was reported that NQO2 activity contributed to the proliferation of A549 and H1299 cancer cells [13], promoted the establishment of bone metastases of LNCaP-C4-2B prostate cancer [14] and indirectly influenced the activation of NF-kB which resulted in the suppression of cell apoptosis and protection of cancer cells against chemotherapy [15], so NQO2 is considered to be a potential anticancer drug target [16,17,18]. At the same time due to its high expression in cancerous cells, NQO2 can be used as an antitumor prodrug activator [19] and can act as an endogenous cancer suppressor [20,21]. The development of novel NQO2 inhibitors may elucidate the role of this enzyme in cancer pathogenesis.

Owing to the extensive research of Boutin and his team, it is generally assumed that NQO2 is the third melatonin binding site, MT3 [22]. Melatonin was able to inhibit NQO2 in the 50 μM range and it was speculated that melatonin antioxidant activity is at least partly a result of this interaction [23]. The majority of potent NQO2 inhibitors contain at least two or three fused aromatic rings, since it advantageous to possess a planar structure capable of engaging in π–π stacking interactions with the cofactor in the active site of the protein. Other known NQO2 ligands include flavonoids [5], ammosamide B analogues [24], imidazoacridin-6-ones [25], furan-amidines [26], etc.

**Figure 1:**
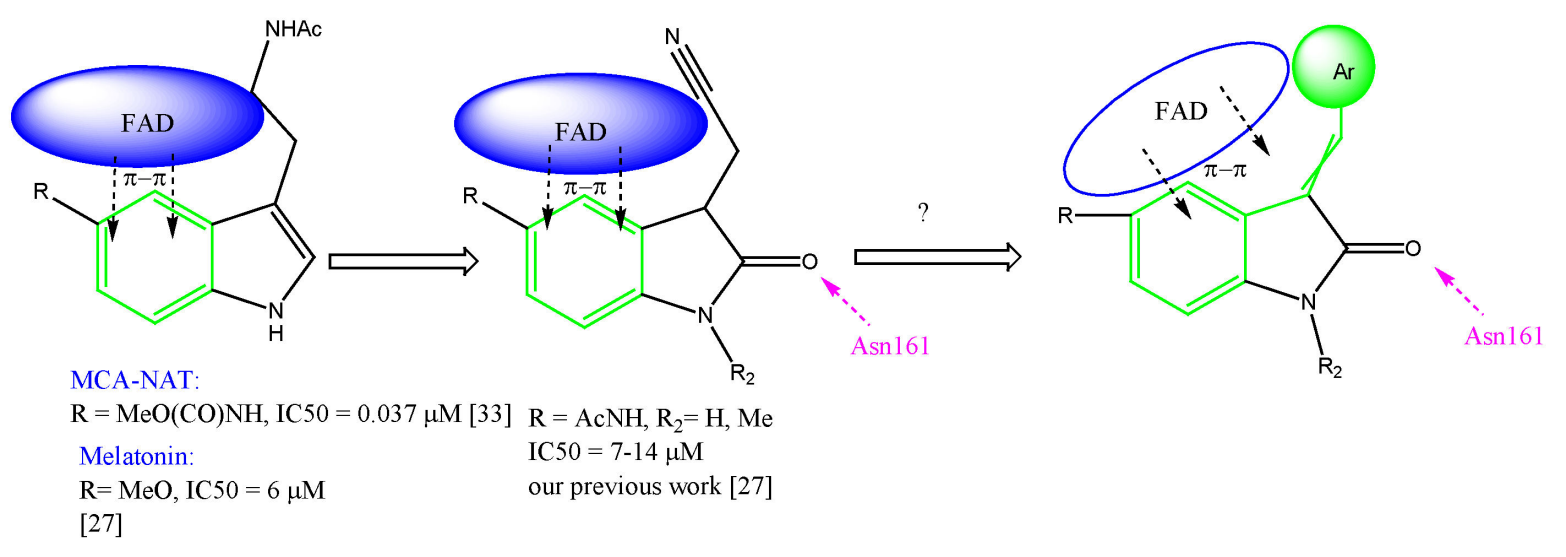
Proposed design of novel oxindole-based NQO2 inhibitors [27].

Previously, we demonstrated that 2-oxindole derivatives can act as NQO2 inhibitors and bind to NQO2 binding site, analogous to melatonin [11,27]. In this work we report the development of new oxindole-based NQO2 inhibitors containing additional arylidene moiety in order to amplify the π–π stacking interactions with the FAD cofactor in the active center of the enzyme (Figure 1).

## 2. Results and Discussion

**Scheme 1:**
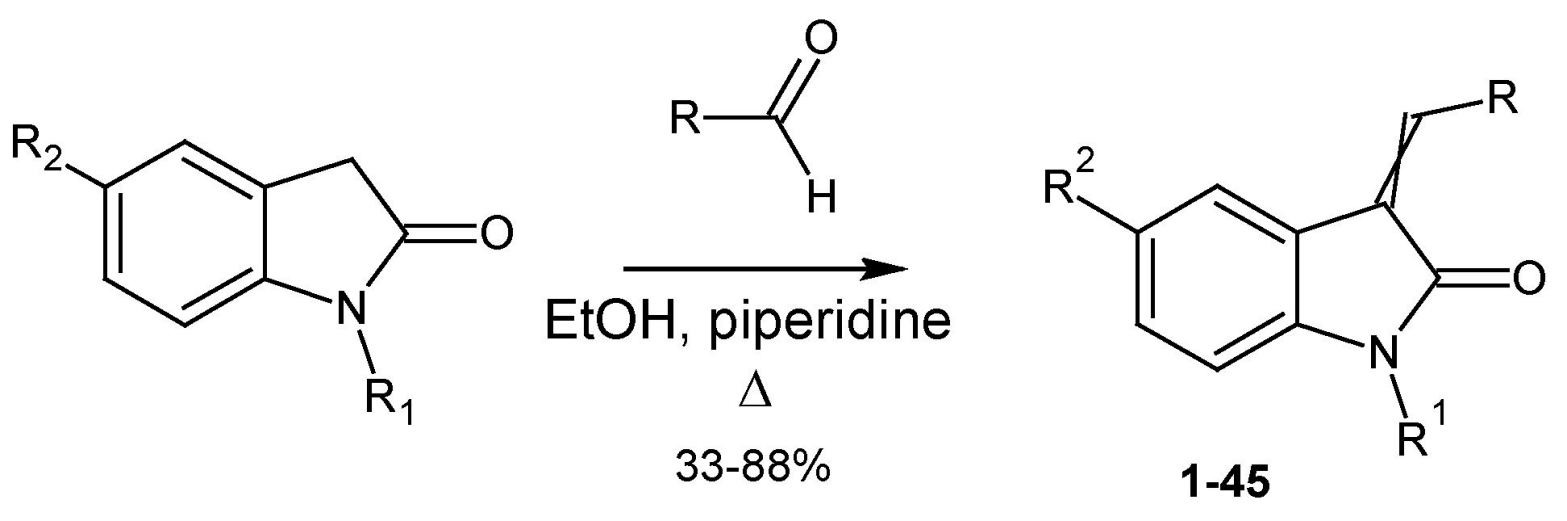
Synthesis of 3-arylidene-2-oxindoles.

To obtain 3-arylidene-2-oxindoles, the following synthetic approach was used (Scheme 1). The aromatic and heteroaromatic aldehydes were condensed with 2-oxindoles in presence of piperidine as a base with good yields. The synthesis of compounds **1**–**4**, **7**–**15**, **17**–**20**, **24**, **25**, **27**–**29**, **31**, **32**, **34**–**44**, **46** and **48**–**52** was performed according to the our previously published procedure [28,29]. Compounds **5**, **6**, **16**, **21**–**23**, **26**, **30**, **33**, **45, 47** were synthesized during this study (Scheme 1).

The detailed structures of the obtained 3-arylidene-2-oxindoles are presented in Table 4.

**Scheme 2:**
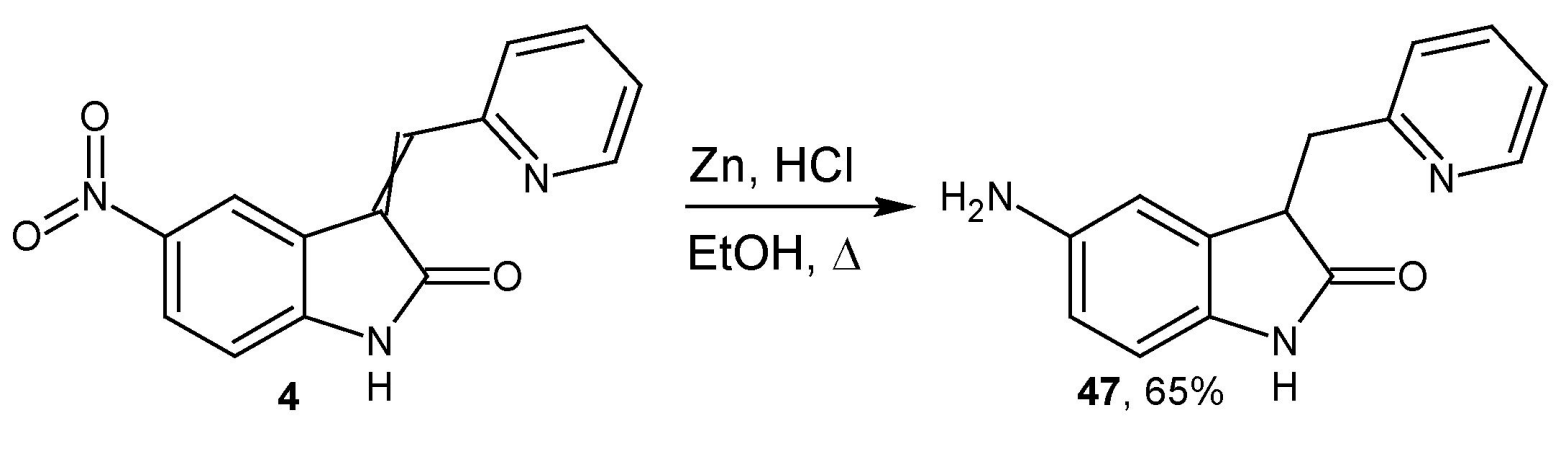
The simultaneous reduction of nitro group and double bond in compound **4**.

The simultaneous reduction of the double bond and nitro group in compound **4** was performed using Zn/HCl (Scheme 2). Compound **47** was prepared analogously [29].

### 2.1. Determining the Configuration of Obtained Compounds

3-arylidene-2-oxindoles were often obtained as mixtures of isomers in various ratios, so it was important to establish convenient criteria for determining the predominant isomer. For several arylidene derivatives, we have identified the characteristic signals in the NMR spectra.

#### 2.1.1. Correlation of 1H NMR Signals for *E*/*Z* Isomers of 4′-Substituted Benzylidene-2-Oxindoles

For some 4′-substituted benzylidene-2-oxindoles, it was previously found that the chemical shift for the 2′(6′) protons of benzylidene moiety in the ^1^H NMR spectra are drastically different for *E*- and *Z*-isomers, being 7.45–7.84 and 7.85–8.53 ppm, respectively [30]. This significant difference exists due to the fact that 2′ and 6′ protons are deshielded by the carbonyl group of oxindole in the case of *Z*-isomers, and at the same time the corresponding protons of the E-isomer are shielded by the benzene ring of the oxindole core structure.

In order to determine the main configuration of our products, we analyzed the NMR spectra of the obtained 4′-substituted benzylidene-2-oxindoles (Table 1). We can confirm that this method for establishing the configuration is very convenient since the characteristic signals in Z-isomers significantly shift downfield and can be easily distinguished from other aromatic signals.

#### 2.1.2. Correlation of 1H NMR Signals for *E*/*Z* Isomers of 3-(Pyridin-2-Ylmethylidene)-2-Oxindoles

**Figure 2:**
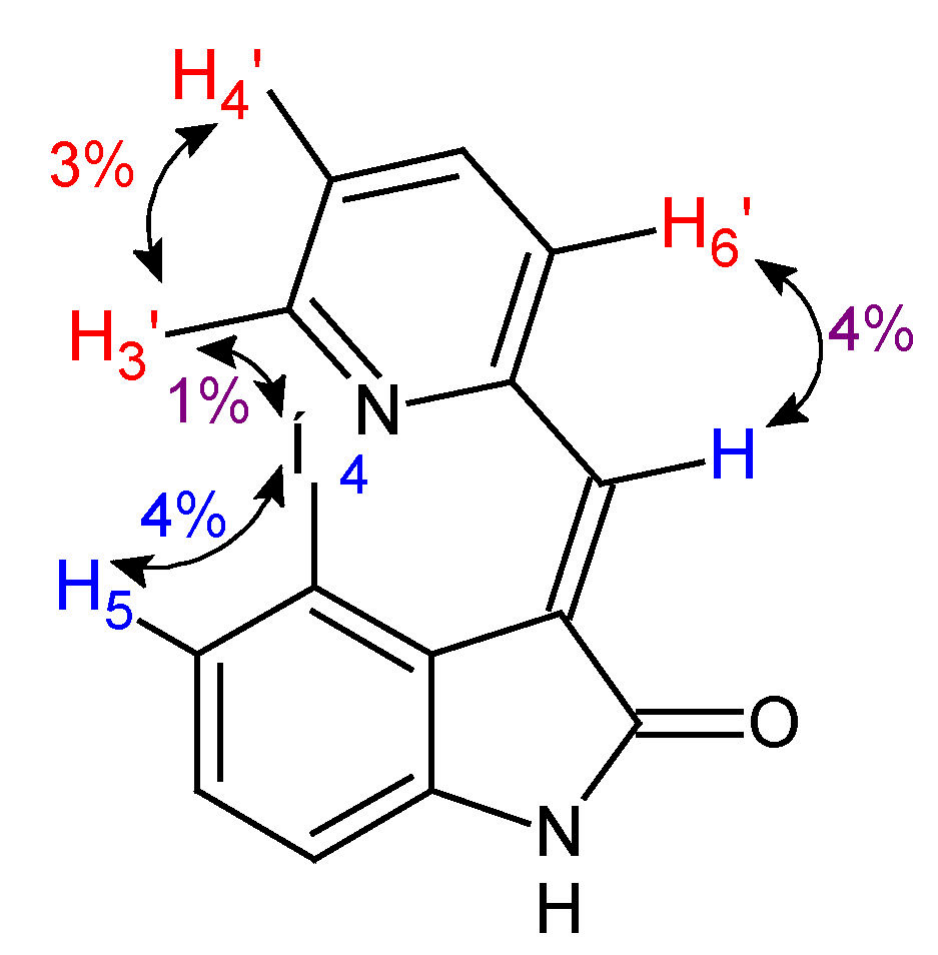
NOE correlations for *E*-isomer of compound **1**.

We found the similar spectral trend for 3-(pyridin-2-ylmethylidene)-substituted oxindole derivatives. In *E*-isomers, the 1H NMR signal which belongs to the H_4_ proton of the oxindole core has a significant downfield shift because of the deshielding by a nearby pyridine nitrogen. To confirm this hypothesis, we carried out an NOE experiment on compound **1** (Figure 2). This experiment confirmed the assignment of the downfield signal to the H_4_ proton via its interaction with the other protons of the oxindole core. The NOE correlation was observed between the downshifted signal of H_4_ and H_3_′ of the pyridine ring, but no interaction was detected between the methylene proton H and the H_4_ oxindole proton. Thus, we have shown that the structure of compound **1**, which was previously incorrectly identified as *Z* [28], actually has the *E* configuration.

A pair of doublets (H_4_ and pyridine H_3′_ signals) located downfield turned out to be a distinguishing feature of (*E*)-3-(pyridin-2-ylmethylidene)-2-oxindole derivatives. Thus, we carried out the assignment of signals for all the obtained compounds and identified the predominant isomer in all cases (Table 2).

#### 2.1.3. Correlation of 1H NMR Signals for E/Z Isomers of Pyrazole Derivatives

**Figure 3:**
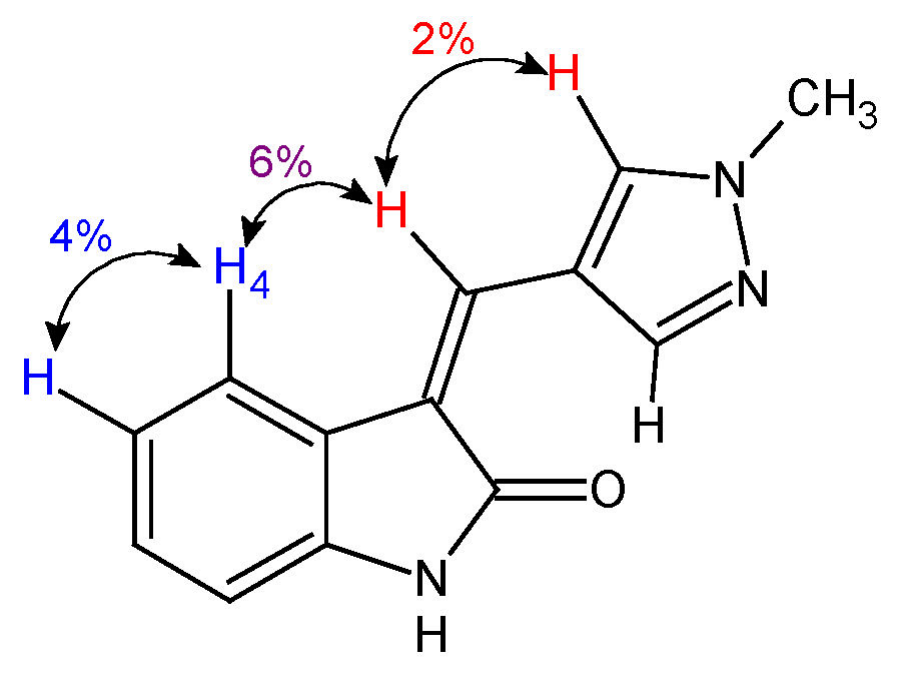
NOE correlation for *Z*-isomer of compound **41**.

We found *Z*-isomer to be predominant in some pyrazole derivatives. The NOE experiment for 3-(1-methyl-*1H*-pyrazol-4-ylmethylidene)-2-oxindole **41**, obtained as a single isomer, showed the interaction of the double bond proton with the proton H_4_ of the oxindole fragment (Figure 3), indicating their *cis* configuration.

The configurations of pyrazole derivative **45** was assigned by comparison of its 1H NMR spectrum with one of the reference compounds. The spectral characteristics of the aromatic region of the 1H NMR spectrum of **45** suggested the predominance of the *Z*-isomer in this compound as well.

### 2.2. The Influence of the Reaction Conditions on Isomer Ratio

As can be seen from the above studies, usually one isomer predominates in the synthesized oxindoles. Since *E*- and *Z*-isomers may have different biological activities, we searched for new reaction conditions that could increase the content of the minor isomer. We investigated the solvent effect and the influence of microwave activation (MW) on the yields and isomeric composition of the products. The reactions were carried out using standard thermal activation and a standard protic solvent (ethanol), an aprotic low-polar solvent (dioxane) and an aprotic polar solvent (ethyl acetate). As model experiments, both reactions with donor and acceptor aldehydes were carried out (Table 3).

**Scheme 3:**
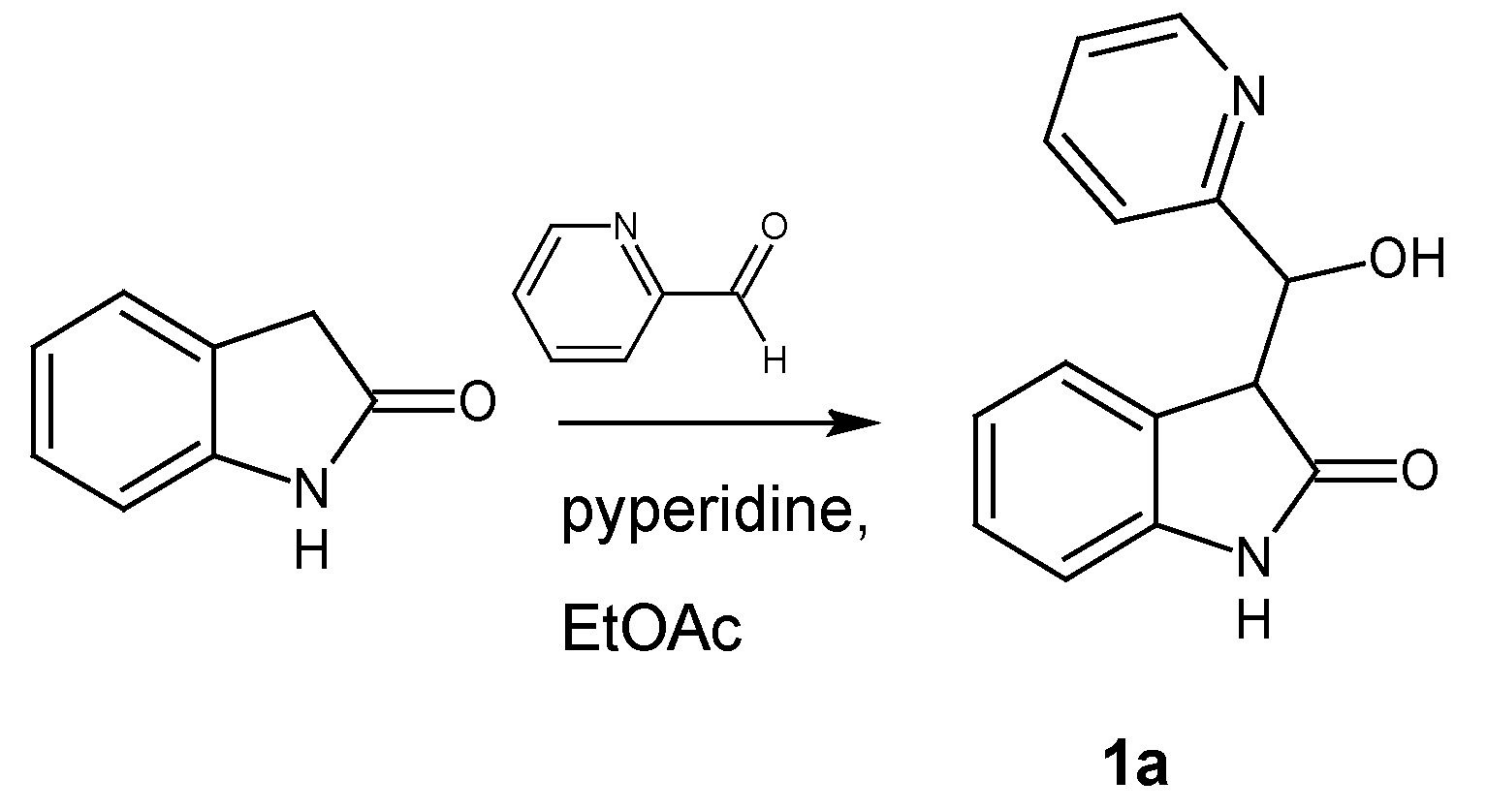
The isolation of intermediate product **1a** using ethyl acetate as solvent.

It is interesting that in case of pyrazole derivatives **39** and **44**, the ratio of isomers in all conditions indicated in Table 3 was not dependent on solvent or activation method while for both 6-membered aryls, the quantity of the minor *Z*-isomer increased with changing ethanol to an aprotic solvent (Table 3). When carrying out the reaction with an acceptor aldehyde in an aprotic solvent, intermediate **1a** precipitated after 10 min and can be isolated. Interestingly, in the case of dioxane, the reaction went further until complete conversion to compound **1** as a mixture of isomers, while in ethyl acetate the reaction stopped at the stage of formation of product **1a** (Scheme 3).

Thus, for non-pyrazole aldehydes, changing the solvent and MW can increase the yield of the minor *Z*-isomer, but does not lead to a complete inversion of the isomeric composition of the products. To obtain the *E*-isomer, standard reaction conditions remain preferable.

### 2.3. Biological Activity of the Obtained Compounds

The inhibition activity of all synthesized compounds at a concentration of 10 µM was preliminarily tested in vitro using human recombinant NRH:quinone oxidoreductase (NQO2). For active compounds, IC_50_ values were also determined. Quercetin [31,32] and melatonin [22] were used as positive controls (Table 4, Figure S21). It was found that the presence of a 3-arylidene moiety was necessary for NQO2 inhibition, while 3-alkyl derivatives were essentially inactive. The most active compounds contain an OH-group in the arylidene moiety (**13**, **15**–**18**). Addition of a 5-acylamino or 5-carbamoylamino group may increase the compound’s inhibition activity, and an increase in affinity was also caused by the addition of a methyl group to the amide nitrogen of the oxindole ring (as demonstrated by compounds **1** and **2** or **14** and **15**).

**Figure 4:**
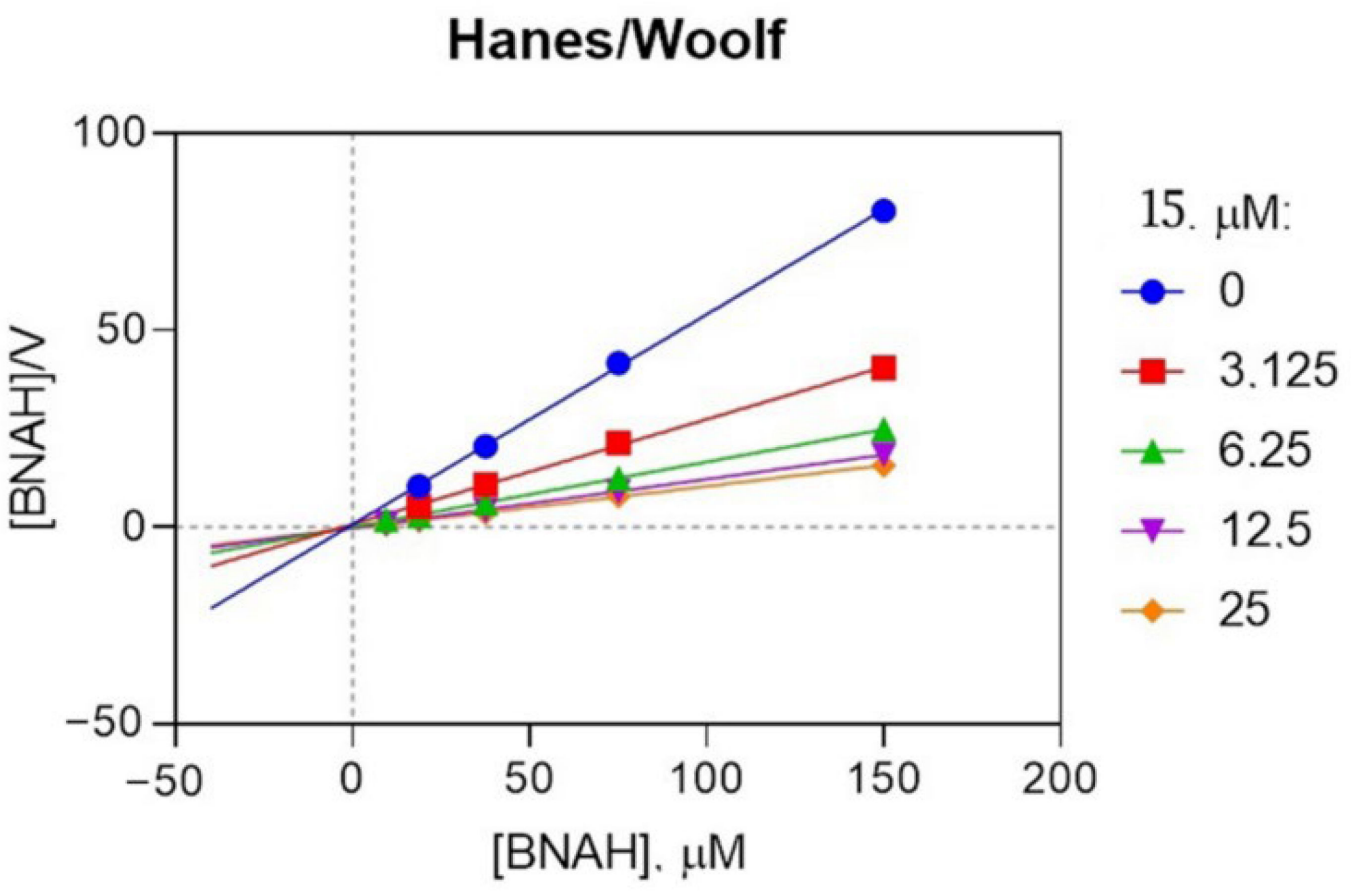
Kinetic evaluation of NQO2 inhibition by compound **15**.

The mechanism of NQO2 inhibition by one of the lead compounds, **15**, was elucidated using a kinetic experiment (Figure 4 and Figure S22). The reaction rate was monitored under a range of inhibitor (0–25 µM) and BNAH substrate concentrations (9.375–150 µM). We found that both maximum rate *V*_max_ and Michaelis constant *K*_m_ decreased while *V*_max_/*K*_m_ ratio increased at higher BNAH concentrations. Hence, **15** behaves as a typical mixed-type (noncompetitive) inhibitor.

The cytotoxicity of the synthesized oxindoles was studied using the A549 cell line in which the levels of NQO2 are increased (Table 4). We found that some oxindoles had moderate cytotoxic effects in the micromolar range of concentration, but no correlation between NQO2 inhibition activity and influence on cell viability was observed. The cytotoxicity of the compounds may be due to the inhibition of other enzymes associated with the activation of apoptosis in cancer cells, for example GSK3b or tyrosine kinases, or with the general non-specific toxicity of these compounds. Thus, the influence of NQO2 on A549 cell viability has not been established.

### 2.4. In Silico Studies

**Figure 5:**
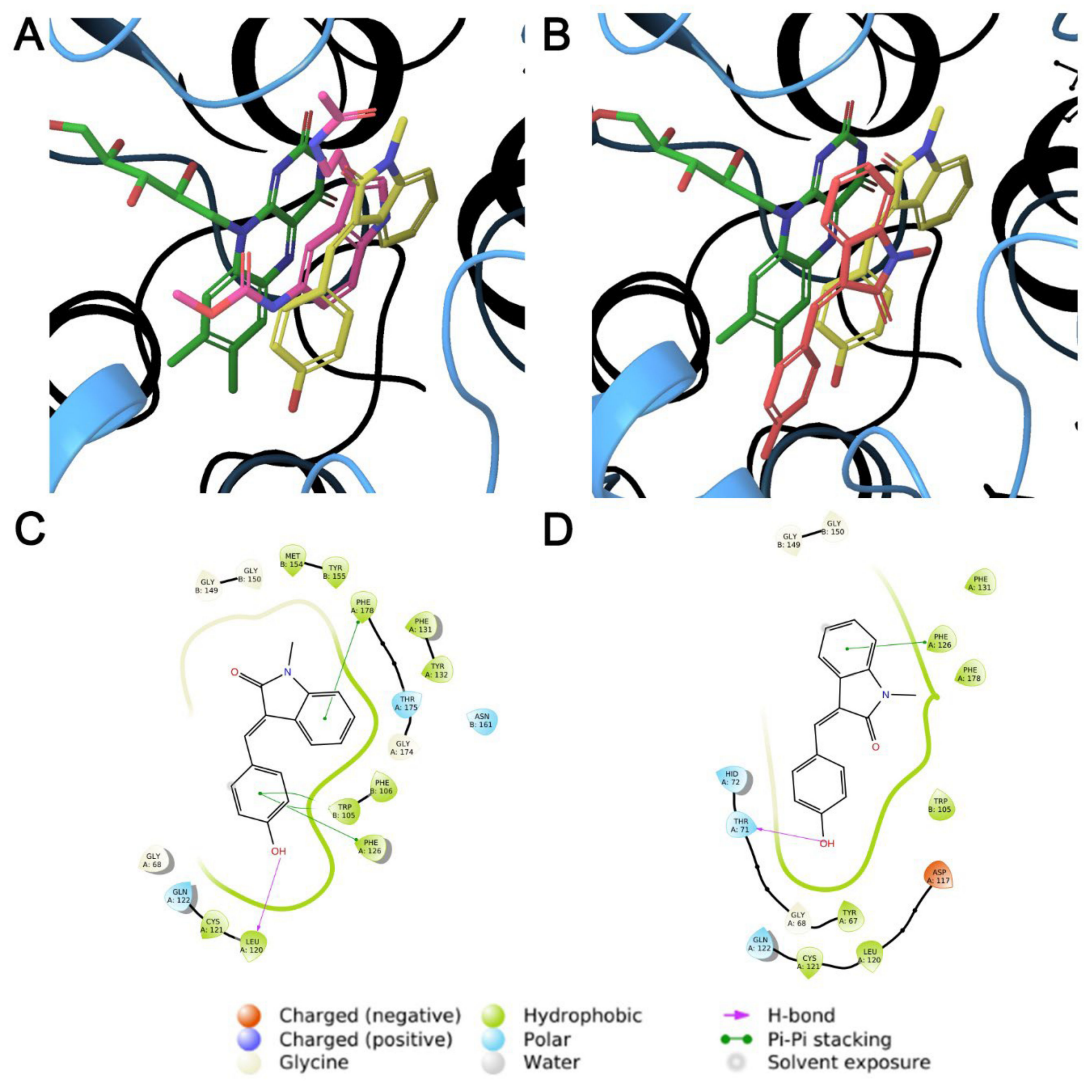
Proposed binding models of MCA-NAT and **15** to active site of NQO2 (blue) with FAD (green). (**A**): binding pose of MCA-NAT (purple) and *E*-isomer (yellow) of **15**. (**B**): *E*- (yellow) and *Z*- (red) isomers of **15**. (**C**): interaction map for the *E*-isomer of **15**. (**D**): interaction map for the *Z*-isomer of **15**.

To analyze the mode of binding of the obtained oxindole derivatives to the NQO2 active site, we performed ligand–protein (Induced Fit) calculations using the Schrodinger software package. The 3OWH PDB structure [33] was used as a protein model. The calculation results are given for active compound **15** (Figure 5), and similar patterns can be traced for the other derivatives (see Supplementary Materials, Figure S23 and Table S1).

The possibility of π–π stacking of both *E*- and *Z*-isomers of **15** with FAD are shown. For the E-isomer, the binding geometry (the first calculated positions according to the docking score) was extremely similar to MCA-NAT (Figure 5A) (PDB 3OVM [33]) and to our previously reported 2-oxindole analogue [27]. The position of the Z-isomer was similar to the E one and to the classic inhibitors mentioned above (Figure 5B). This allows us to conclude that there is a possibility of binding both isomers (*Z* and *E*) in a manner similar to MCA-NAT.

## 3. Materials and Methods

### 3.1. Chemistry

All solvents were used as received without further purification. The reactions were monitored by thin layer chromatography (TLC) carried out on Merck TLC silica gel plates (60 F254), using UV light for visualization. Flash column chromatography purifications were carried out using silica gel 60 (particle size 0.040–0.060 mm).

^1^H and ^13^C NMR spectra were recorded at 298 K on a Bruker Avance 300 spectrometer with operating frequencies of 400.13 and 100.6 MHz, respectively, and calibrated using residual CHCl_3_ (δH = 7.26 ppm) and CDCl_3_ (δC = 77.16 ppm) or DMSO-d_5_ (δH = 2.50 ppm) and DMSO-*d_6_* (δC = 39.52 ppm) as internal references. NMR data were presented as follows: chemical shift (δ ppm), multiplicity (s = singlet, d = doublet, dd = doublet of doublets, t = triplet, td = triplet of doublets, q = quartet, m = multiplet, br. = broad), coupling constant (J) in Hertz (Hz), integration. High-resolution mass spectra (HRMS) were measured on a Thermo Scientific LTQ Orbitrap instrument using nanoelectrospray ionization (nano-ESI). The reactions under microwave irradiation were carried out in a Monowave 300 microwave reactor (Anton Paar, GmbH, Graz, Austria), as well as in a Bosch HMT72M420 domestic microwave oven with a volume of 17 L. The reactions under thermal activation conditions were carried out on laboratory hot plates, as well as in a ChemiStation chemical reactor (EYELA). ^1^H and ^13^C NMR spectra of all novel compounds (Figures S1–S20) are given in Supplementary Materials.

Isatin, 5-methoxyisatin and 5-bromoisatin were purchased from Merck. *N*-methyloxindole was obtained by *N*-alkylation using a previously described procedure [34]. The following compounds were obtained as previously described: 5-nitro-2-oxindole [28], 5-amino-2-oxindole [28], 5-acetamido-2-oxindole [28], 5-methoxycarbonylamino-2-oxindole [28], 5-benzoylamino-2-oxindole [28], 5-furoylamino-2-oxindole [28], (*E*)-3-(2-pyridinylmethylidene)-2-oxindole 1 [28], *N*-methyl-3-((pyridin-2-yl)methylene)-2-oxindole 2 [29], 5-bromo-3-(2-pyridinylmethylidene)-2-oxindole 3 [28], 5-nitro-3-(2-pyridinylmethylidene)-2-oxindole 4 [28], 3-(3-pyridinylmethylidene)-2-oxindole 7 [28], 5-bromo-3-(3-pyridinylmethylidene)-2-oxindole 8 [28], 5-nitro-3-(3-pyridinylmethylidene)-2-oxidole 9 [28], 3-(4-pyridinylmethylidene)-2-oxindole 10 [28], 5-bromo-3-(4-pyridinylmethylidene)-2-oxindole 11 [28], 5-nitro-3-(4-pyridinylmethylidene)-2-oxindole 12 [28], 3-(3-hydroxybenzylidene)-5-acetylamino-2-oxindole 13 [28], 3-(4-hydroxybenzylidene)-2-oxindole 14 [29], 3-(4-hydroxybenzylidene)-N-methyl-2-oxindole 15 [29], 3-(4-hydroxybenzylidene)-5-(methoxycarbonylamino)-2-oxindole 17 [28], 3-(4-hydroxybenzylidene)-5-furoylamino-2-oxindole 18 [28], 3-(4-hydroxybenzylidene)-5-benzoylamino-2-oxindole 19 [28], 3-((4-methoxyphenyl)methylene)-2-oxindole 20 [29], 3-(3,4,5-trimethoxybenzylidene)-5-acetylamino-2-oxindole 24 [28], 3-(3,4,5-trimethoxybenzylidene)-5-furoylamino-2-oxindole 25 [28], 3-((4-nitrophenyl)methylene)-2-oxindole 27 [29], 3-(4-nitrobenzylidene)-5-(methoxycarbonylamino)-2-oxindole 28 [28], 5-benzoylamino-3-((4-nitrophenyl)methylene)-2-oxindole 29 [29], 3-(4-bromobenzylidene)-5-bromo-2-oxindole 31 [28], 3-(4-bromobenzylidene)-5-(methoxycarbonylamino)-2-oxindole 32 [28], 3-(2-furylmethylidene)-2-oxindole 34 [28], 5-bromo-3-(2-furylmethylidene)-2-oxindole 35 [28], 5-nitro-3-(2-furylmethylidene)-2-oxindole 36 [28], 3-(2-thienylmethylidene)-2-oxindole 37 [28], 5-bromo-3-(2-thienylmethylidene)-2-oxindole 38 [28], 3-((5-ethoxycarbonyl-1H-pyrazol-4-yl)methylene)-2-oxindole 39 [29], 3-((5-ethoxycarbonyl-1H-pyrazol-4-yl)methylene)-5-nitro-2-oxindole 40 [29], 3-((1-methyl-1H-pyrazol-4-yl)methylene)-2-oxindole 41 [29], 3-((3,5-dimethyl-1H-pyrazol-4-yl)methylene)-2-oxindole 42 [29], 3-((3-methyl-1H-pyrazol-4-yl)methylene)-2-oxindole 43 [29], 3-((3,5-dimethyl-1-phenyl-1H-pyrazol-4-yl)methylene)-2-oxindole 44 [29], 3-(4-aminobenzyl)-2-oxindole 46 [29], 3-hydroxy-5-methoxy-3-[2-(4-methylphenyl)-2-oxoethyl]-2-oxindole 48 [28], 3-hydroxy-5-bromo-3-[2-(4-methylphenyl)-2-oxoethyl]-2-oxindole 49 [28], 3-hydroxy-5-bromo-3-[2-(4-methoxyphenyl)-2-oxoethyl]-2-oxindole 50 [28], 3-hydroxy-5-(2-furoylamino)-3-[2-(4-methylphenyl)-2-oxoethyl]-2-oxindole 51 [28], 3-hydroxy-3-[2-(4-methylphenyl)-2-oxoethyl]-2-oxindole 52 [28].

#### 3.1.1. General Procedure for Synthesis of 3-Arylidene-2-Oxindoles

2-oxindole (1 eq.) and the corresponding carboxaldehyde (1 eq.) were dissolved in 2–5 mL of solvent and 60–120 µL of piperidine was added. The reaction mixture was refluxed until the parent oxindole disappeared (monitored by TLC). The reaction mixture was then cooled down to room temperature. If a precipitate formed, it was filtered off and washed with ethyl acetate and diethyl ether and dried. If no precipitate appeared, the solution was concentrated in a vacuum and the resulting product was washed with ethyl acetate and diethyl ether and dried. The following compounds were obtained according to this procedure, with the reactions carried out in ethanol:

(*E*,*Z*)-3-(2-Pyridinylmethylidene)-5-acetamido-2-oxindole **5**
From 0.18 g (0.94 mmol) of 5-acetamido-2-oxindole, 87 µL (0.94 mmol) of 2-pyridinecarboxaldehyde and 75 µL (0.88 mmol) of piperidine with ethanol as solvent the reddish-brown powder (0.156 g, 59%) was obtained as a mixture of two isomers. According to ^1^H NMR *E*/*Z* stereoisomer ratio is 7.15:1, respectively.
*E* isomer, ^1^H NMR (400.13 MHz, DMSO-*d_6_*, ppm): 2.02 (s, 3H, CH_3_), 6.76 (d, *J* = 8.4 Hz, 1H, H_7_), 7.42–7.48 (m, 1H), 7.49–7.55 (m, 2H), 7.82 (d, *J*= 7.7 Hz, 1H), 7.92 (td, *J*_1_ = 7.7 Hz, *J*_2_ = 1.8 Hz, 1H), 8.90 (d, *J* = 3.7 Hz, 1H, H_3′_), 9.30 (d, *J* = 2.0 Hz, 1H, H_4_), 9.89 (br.s, 1H, NH), 10.53 (br. s, 1H, NH).
Selected peaks of *Z* isomer, ^1^H NMR (400.13 MHz, DMSO*-d_6_*, ppm): 1.96 (s, 3H, CH_3_), 6.85 (d, *J* = 7.9 Hz, 1H, H_7_), 7.66–7.71 (m, 1H), 8.00–8.05 (m, 1H), 8.80 (d, *J* = 4.8 Hz, 1H, H_3′_).
^13^C NMR (100.6 MHz, DMSO-*d_6_*, ppm): 24.35, 109.61, 120.54, 121.91, 122.44, 124.45, 128.47, 130.18, 133.79, 134.19, 137.61, 139.66, 150.16, 153.59, 168.25, 169.77.
HRMS (ESI), *m*/*z*: (M+H) found 280.1073, C_16_H_14_N_3_O_2_ requires 280.1086, (M + Na) found 302.0891, C_16_H_13_N_3_O_2_Na requires 302.0905.
(*E*,*Z*)-3-(2-Pyridinylmethylidene)-5-benzoylamino-2-oxindole **6**
From 0.100 g (0.4 mmol) of 5-benzoylamino-oxindole, 43 µL (0.4 mmol) of 2-pyridinecarboxaldehyde and 30 µL (0.35 mmol) of piperidine with ethanol as solvent the light brown powder (0.084 g, 62%) was obtained as a mixture of two isomers. According to ^1^H NMR *E*/*Z* stereoisomer ratio is 6:1, respectively.
*E* isomer, ^1^H NMR (400.13 MHz, DMSO-d_6_, ppm): 6.84 (d, J = 8.4 Hz, 1H), 7.41–7.58 (m, 5H), 7.61 (dd, J_1_ = 8.4 Hz, J_2_ = 2.0 Hz, 1H), 7.84 (d, J = 7.7 Hz, 1H), 7.88–7.97 (m, 4H), 8.88 (d, J = 3.9 Hz, 1H, H_3′_), 9.46 (d, J = 1.7 Hz, 1H, H_4_), 10.22 (br. s, 1H, NH), 10.67 (br.s, 1H, NH).
Selected peaks of Z isomer, ^1^H NMR (400.13 MHz, DMSO-d_6_, ppm): 7.02 (d, J = 8.8 Hz, 1H, H_7_), 7.19–7.30 (m, 3H), 7.72 (d, J = 7.7 Hz, 1H).
^13^C NMR (100.6 MHz, DMSO-d_6_, ppm): 109.27, 121.50, 121.68, 123.85, 124.12, 127.69, 128.14, 128.37, 129.74, 131.35, 132.98, 133.93, 135.30, 137.24, 139.97, 149.83, 153.20, 165.37, 169.49.
HRMS (ESI), *m*/*z*: (M + H) found 342.1233, C_21_H_16_N_3_O_2_ requires 342.1242, (M + Na) found 364.1051, C_21_H_15_N_3_O_2_Na requires 364.1062, (M+K) found 380.0790, C_21_H_15_N_3_O_2_K requires 380.0801.
(*E*,*Z*)-3-(4-Hydroxybenzylidene)-5-acetamido-2-oxindole **16**
From 0.192 g (1 mmol) of 5-acetamido-2-oxindole, 0.122 g (1 mmol) of 4-hydroxybenzaldehyde and 80 µL (0.94 mmol) of piperidine with ethanol as solvent the yellow powder (0.189 g, 64%) was obtained as a mixture of two isomers. According to ^1^H NMR E/Z stereoisomer ratio is 2.6:1 respectively.
E isomer, ^1^H NMR (400.13 MHz, DMSO-d_6_, ppm): 1.99 (s, 3H, CH_3_), 6.78 (d, J = 8.3 Hz, 1H, H_7_), 6.87 (d, J = 8.6 Hz, 2H, H_3′_,H_5′_), 7.40 (td, J_1_ = 8.3 Hz, J_2_ = 1.9 Hz, 1H, H_6_), 7.51 (s, 1H, CH=), 7.62 (d, J = 8.7 Hz, 2H, H_2′_,H_6′_), 8.12 (d, J = 1.9 Hz, 1H, H_4_), 9.80 (s, 1H, NH), 10.42 (br.s, 1H, NH).
Selected peaks of Z isomer, ^1^H NMR (400.13 MHz, DMSO-d_6_, ppm): 2.02 (s, 3H, CH_3_), 6.70–6.83 (m, 3H), 7.22 (dd, J_1_ = 8.3 Hz, J_2_ = 1.9 Hz, 1H, H_6_), 7.48 (s, 1H, CH=), 7.82 (d, J = 1.9 Hz, 1H, H_4_), 8.39 (d, J = 8.8 Hz, 2H, H_2′_,H_6′_), 9.68 (s, 1H, NH), 10.46 (br.s, 1H, NH).
^13^C NMR (100.6 MHz, DMSO-d_6_, ppm): 24.29, 24.49, 109.98, 114.47, 116.11, 116.46, 120.83, 121.84, 124.45, 124.58, 132.57, 133.56, 135.58, 137.40, 138.44, 161.08, 168.27, 169.79.
(*E*,*Z*)-3-(4-Methoxybenzylidene)-5-benzoylamino-2-oxindole **21**
From 0.100 g (0.4 mmol) of 5-(benzoylamino)oxindole, 0.055 g (0.4 mmol) of 4-methoxybenzaldehyde and 30 µL (0.35 mmol) of piperidine with ethanol as solvent the greenish grey powder (0.113 g, 77%) was obtained as a mixture of two isomers. According to ^1^H NMR E/Z stereoisomer ratio is 2:1, respectively.
E isomer, ^1^H NMR (400.13 MHz, DMSO-d_6_, ppm): 3.81 (s, 3H, CH_3_), 6.88 (d, J = 8.3 Hz, 1H, H_7_), 7.07 (d, J = 8.7 Hz, 2H), 7.43–7.66 (m, 5H), 7.76 (d, J = 8.3 Hz, 2H), 7.91 (d, J = 7.2 Hz, 2H), 8.24 (s, 1H, CH=), 10.18 (br.s, 1H, NH), 10.60 (br.s, 1H, NH).
Selected peaks of Z isomer, ^1^H NMR (400.13 MHz, DMSO-*d*_6_, ppm): 3.82 (s, 3H, CH_3_), 6.82 (d, J = 8.4 Hz, 1H, H_7_), 7.03 (d, J = 8.6 Hz, 2H), 7.86 (d, J = 8.6 Hz, 1H, H_ind_), 8.00 (d, J = 7.1 Hz, 2H), 8.09 (s, 1H, CH=), 8.51 (d, J = 8.7 Hz, 2H, H_2′_,H_6′_), 9.85 (br.s, 1H, NH), 10.22 (br.s, 1H, NH).
^13^C NMR (100.6 MHz, DMSO-d_6_, ppm): 55.40, 109.72, 113.80, 114.38, 115.63, 121.12, 121.85, 122.59, 124.28, 125.29, 125.54, 126.53, 126.92, 127.59, 127.64, 128.36, 128.42, 131.41, 131.47, 131.79, 131.85, 132.84, 132.88, 134.59, 135.12, 136.24, 136.85, 136.88, 138.94, 160.61, 161.29, 165.24, 165.36, 167.62, 169.24.
(*E*)-3-(4-Ethoxybenzylidene)-2-oxindole **22**
From 0.100 g (0.8 mmol) of 2-oxindole, 0.115 g (0.8 mmol) of 4-ethoxybenzaldehyde and 60 µL (0.7 mmol) of piperidine with ethanol as solvent the yellow powder (0.146 g, 73%) was obtained as a single *E* isomer.
m.p. = 170–171 °C (m.p.^lit.^ 169–171 [35])
^1^H NMR (400.13 MHz, DMSO-*d_6_*, ppm): 1.35 (t, *J* = 7.0 Hz, 3H, CH_3_), 4.07–4.14 (m, 2H, CH_2_), 6.83–6.89 (m, 2H, H_7_,H_ind_), 7.06 (d, *J* = 8.7 Hz, 2H, H_3′_,H_5′_), 7.21 (td, *J*_1_ = 7.0 Hz, *J*_2_ = 0.7 Hz, 1H, H_ind_), 7.56 (s, 1H, CH=), 7.65 (d, *J* = 7.8 Hz, 1H, H_4_), 7.69 (d, *J* = 8.7 Hz, 2H, H_2′_,H_6′_), 10.55 (br.s, 1H, NH).
(*E*,*Z*)-3-(3,4,5-Trimethoxybenzylidene)-2-oxindole 23
From 0.100 g (0.8 mmol) of 2-oxindole, 0.143 g (0.8 mmol) of 3,4,5-trimethoxybenzaldehyde and 60 µL (0.7 mmol) of piperidine with ethanol as solvent the yellow powder (0.187 g, 84%) was obtained as a mixture of two isomers. According to ^1^H NMR stereoisomer ratio is 1.66:1. [36]
Major isomer ^1^H NMR (400.13 MHz, DMSO-*d_6_*, ppm): 3.89 (s, 6H, CH_3_), 3.95 (s, 3H, CH_3_), 6.86–6.95 (m, 2H, H_ind_), 7.24 (t, *J* = 7.6 Hz, 1H, H_ind_), 7.27 (s, 2H, H_2′_,H_6′_), 7.79–7.83 (m, 2H, H_ind_,CH=), 8.13 (br.s, 1H, NH).
Selected peaks of minor isomer ^1^H NMR (400.13 MHz, DMSO-*d_6_*, ppm): 3.98 (s, 9H, CH_3_), 6.88 (d, *J* = 7.7 Hz, 1H, H_7_) 7.06 (t, *J* = 7.4 Hz, 1H, H_ind_), 7.49 (s, 1H, CH=), 7.54 (d, *J* = 7.5 Hz, 1H, H_4_), 8.02 (br.s, 1H, NH)
(*E*,*Z*)-3-(3,5-Dimethoxy-4-hydroxybenzylidene)-5-benzoylamino-2-oxindole **26**
From 0.100 g (0.4 mmol) of 5-benzoylamino-oxindole, 0.089 g (0.4 mmol) of 3,5-dimethoxy-4-hydroxybenzaldehyde and 30 µL (0.35 mmol) of piperidine with ethanol as solvent the black powder (0.133 g, 79%) was obtained as a mixture of two isomers. According to ^1^H NMR stereoisomer ratio is 1.66:1.
Major isomer, ^1^H NMR (400.13 MHz, DMSO-d_6_, ppm): 3.78 (s, 6H, CH_3_), 5.04 (br.s, 1H, OH), 6.83 (d, J = 8.3 Hz, 1H, H_7_), 7.05 (s, 2H, H_2′_,H_6′_), 7.37–7.59 (m, 4H), 7.87 (d, J = 7.2 Hz, 2H, H_Ar_), 8.06 (s, 1H, CH=), 8.52 (br.s, 1H, H_4_), 9.51 (br.s, 1H, NH), 10.15 (br.s, 1H, NH);
Selected signals of minor isomer, ^1^H NMR (400.13 MHz, DMSO-d_6_, ppm): 3.72 (s, 6H, CH_3_), 6.78 (d, J = 8.3 Hz, 1H, H_7_), 7.01 (s, 2H, H_2′_,H_6′_), 7.91 (s, 1H, CH=), 7.96 (d, J = 7.0 Hz, 2H, H_Ar_), 8.02 (s, 1H, H_4_), 10.13 (br.s, 1H, NH);
^13^C NMR (100.6 MHz, DMSO-d_6_, ppm): 56.08, 56.15, 108.11, 108.45, 109.93, 111.80, 114.39, 122.00, 122.08, 122.93, 123.81, 127.91, 128.76, 128.79, 131.77, 133.27, 135.39, 135.49, 138.22, 139.03, 148.57, 165.66, 169.89.
(*E*,*Z*)-3-(4-Dimethylaminobenzylidene)-2-oxindole **30**
From 0.100 g (0.8 mmol) of 2-oxindole, 0.12 g (0.8 mmol) of 4-(dimethylamino)benzaldehyde and 60 µL (0.7 mmol) of piperidine with ethanol as solvent the reddish-brown powder (0.107 g, 54%) was obtained as a mixture of two isomers. According to ^1^H NMR *E*/*Z* stereoisomer ratio is 1:1 respectively [30].
^1^H NMR (400.13 MHz, DMSO-*d_6_*, ppm): 3.01 (s, 6H, CH_3_), 3.02 (s, 6H, CH_3_), 6.70–6.95 (m, 8H), 7.10 (t, *J* = 7.7 Hz, 1H), 7.17 (t, *J* = 7.8 Hz, 1H), 7.51 (s, 1H, CH=), 7.57–7.67 (m, 4H), 7.78 (d, *J* = 7.5 Hz, 1H, H_4_), 8.44 (d, *J* = 8.7 Hz, 2H, H_2′_,H_6′_)*, 10.54 (br. s, 2H, NH).
*– Z isomer.
(*E*,*Z*)-3-(4-Fluorobenzylidene)-2-oxindole **33**
From 0.100 g (0.8 mmol) of 2-oxindole, 0.95 g (0.8 mmol) of 4-fluorobenzaldehyde and 60 µL (0.7 mmol) of piperidine with ethanol as solvent yellow powder (0.122 g, 68%) was obtained as a mixture of two isomers. According to ^1^H NMR E/Z stereoisomer ratio is 2:1, respectively [37].
E isomer, ^1^H NMR (400.13 MHz, DMSO-d_6_, ppm): 6.78–6.89 (m, 2H, H_7_,H_ind_), 7.17–7.25 (m, 1H, H_ind_), 7.27–7.38 (m, 2H, H_3′_,H_5′_), 7.49 (d, *J* = 7.6 Hz, 1H, H_4_), 7.58 (s, 1H CH=), 7.73–7.75 (m, 2H, H_2′_,H_6′_), 10.62 (br. s 1H, NH).
Selected peaks of Z isomer, ^1^H NMR (400.13 MHz, DMSO-d_6_, ppm): 6.98 (t, J = 7.5 Hz, 1H, H_ind_), 7.69 (d, J = 7.5 Hz, 1H, H_4_), 7.8 (s, 1H, CH=), 8.45–8.5 (m, 2H, H_2′_, H_6′_), 10.66 (br.s, 1H, NH).
(*E*,*Z*)-3-(1-[2-(Methoxycarbonyl)ethyl]-1H-pyrazol-4-ylmethylidene)-2-oxindole **45**
From 0.100 g (0.8 mmol) of 2-oxindole, 0.142 g (0.8 mmol) methyl 3-(4-formyl-*1H*-pyrazol-1-yl)propanoate and 60 µL (0.7 mmol) of piperidine with ethanol as solvent the yellow powder (0.187 g, 84 %) was obtained as a mixture of two isomers. According to ^1^H NMR *E*/*Z* stereoisomer ratio is 1:3.5, respectively.
*Z* isomer, ^1^H NMR (400.13 MHz, DMSO-*d_6_*, ppm): 2.87–2.97 (m, 2H, CH_2_), 3.59 (s, 3H, CH_3_), 4.39–4.49 (m, 2H, CH_2_), 6.81 (d, *J* = 7.6 Hz, 1H, H_7_), 6.91–7.00 (m, 1H, H_ind_), 7.14 (t, *J* = 7.4 Hz, 1H, H_ind_), 7.57 (d, *J* = 7.3 Hz, 1H, H_4_), 7.67 (s, 1H, CH=), 8.24 (s, 1H, CH_pyr_), 8.83 (s, 1H, NH_pyr_), 10.54 (br. s, 1H, NH_ind_).
Selected peaks of *E*-isomer, ^1^H NMR (400.13 MHz, DMSO-*d_6_*, ppm): 2.87–2.97 (m, 2H, CH_2_), 3.59 (s, 3H, CH_3_), 4.39–4.49 (m, 2H, CH_2_), 6.86 (d, *J* = 7.6 Hz, 1H, H_7_), 7.21 (t, *J* = 7.6 Hz, 1H, H_ind_), 7.45 (s, 1H, CH=), 7.82 (d, *J* = 7.4 Hz, 1H), 8.00 (s, 1H), 8.42 (s, 1H), 10.5 (br. s, 1H, NH_ind_).
^13^C NMR (100.6 MHz, DMSO-*d_6_*, ppm): 33.87, 47.31, 51.64, 109.28, 116.98, 118.89, 120.87, 121.77, 124.81, 126.30, 127.82, 134.70, 139.98, 143.53, 167.65, 169.30, 171.10.
HRMS (ESI), *m*/*z*: (M+H) found 298.1178, C_16_H_16_N_3_O_3_ requires 298.1192, (M+Na) 320.0095 C_16_H_15_N_3_O_3_Na requires 320.1011.

#### 3.1.2. Influence of solvent on E/Z Isomer Ratio

The general synthesis procedure was used for the synthesis of followed compounds with some variations: 1,4-dioxane or ethyl acetate was used instead of ethanol.

(*E*)-3-(2-Pyridinylmethylidene)-2-oxindole **(*E*)-1**
From 0.200 g (1.5 mmol) of 2-oxindole, 0.16 mL (1.5 mmol) of 2-pyridinecarboxaldehyde and 120 µL (1.5 mmol) of piperidine with ethanol as solvent the brown powder (0.109 g, 61%) was obtained as a single E isomer [28].
^1^H NMR (400.13 MHz, DMSO-d_6_, ppm): 6.84 (d, J = 7.7 Hz, 1H, H_7_), 6.95 (td, J_1_ = 7.7 Hz, J_2_ = 0.9 Hz, 1H, H_5_), 7.25 (td, J_1_ = 7.5 Hz, J_2_ = 1.3 Hz, H_6_), 7.41 (ddd, J_1_ = 7.5 Hz, J_2_ = 4.8 Hz, J_3_ = 1.1 Hz, 1H, H_4′_), 7.54 (s, 1H, CH=), 7.82 (d, J = 7.5 Hz, 1H, H_6′_), 7.90 (td, J_1_ = 7.7 Hz, J_2_ = 1.8 Hz, 1H, H_5′_), 8.4 (d, J = 3.9 Hz, 1H, H_3′_), 8.98 (d, J = 7.3 Hz, 1H, H_4_), 10.61 (br.s, 1H, NH).
(*E*,*Z*)-3-(2-Pyridinylmethylidene)-2-oxindole **(*E*,*Z*)-1**
From 0.100 g (0.8 mmol) of 2-oxindole, 0.08 mL (0.8 mmol) of 2-pyridinecarboxaldehyde and 60 µL (0.7 mmol) of piperidine with 1,4-dioxane as solvent the brown oil (0.145 g, 81%) was obtained as a mixture of two isomers. According to ^1^H NMR *E*/*Z* stereoisomer ratio is 1.3:1 respectively. ^1^H NMR (DMSO-*d_6_*) for *E* isomer is identical to described above.
Selected signals of Z-isomer, ^1^H NMR (400.13 MHz, DMSO-*d_6_*, ppm): 7.06 (d, *J* = 7.9 Hz, 1H, H_4_), 7.63 (s, 1H, CH=).
3-(Hydroxy(pyridin-2-yl)methyl)-2-oxindole **1a**
From 0.100 g (0.8 mmol) of 2-oxindole, 0.08 mL (0.8 mmol) of 2-pyridinecarboxaldehyde and 60 µL (0.7 mmol) of piperidine with ethyl acetate as solvent the beige powder (0.088 g, 49%) was obtained. According to ^1^H NMR the ratio of two diastereoisomers of 1a and the final condensation product 1 is 6.92:4.17:1 respectively.
Major isomer, ^1^H NMR (400.13 MHz, DMSO-d_6_, ppm): 4.08 (d, J = 2.4 Hz, 1H), 5.33 (dd, J_1_ = 4.7 Hz, J_2_ = 1.9 Hz, 1H), 6.62 (t, J = 7.6 Hz, 1H), 6.77 (d, J = 7.7 Hz, 1H), 7.02–7.10 (m, 1H), 7.34 (dd, J_1_ = 7.2 Hz, J_2_ = 2.2 Hz, 1H), 7.51 (d, J = 7.8 Hz, 1H), 7.85 (td, J_1_ = 7.7 Hz, J_2_ = 1.6 Hz, 1H), 8.62 (d, J = 4.2 Hz, 1H), 10.39 (br.s, 1H)
Selected peaks of minor isomer ^1^H NMR (400.13 MHz, DMSO-d_6_, ppm): 3.96 (d, J = 3.0 Hz, 1H), 5.27 (t, J = 3.5 Hz, 1H), 6.67 (d, J = 7.7 Hz, 1H), 6.83 (t, J = 7.5 Hz, 1H), 7.13 (dd, J_1_ = 7.1 Hz, J_2_ = 1.8 Hz, 1H), 7.25 (d, J = 7.3 Hz, 1H), 7.42 (d, J = 8.0 Hz, 1H), 7.66 (td, J_1_ = 7.8 Hz, J_2_ = 1.7 Hz, 1H), 8.35 (d, J = 4.1 Hz, 1H), 10.17 (br.s, 1H).
1H spectrum also contains signals of 1.
^13^C NMR (100.6 MHz, DMSO-d_6_, ppm): 57.07, 57.29, 77.74, 78.66, 113.84, 114.22, 125.66, 125.79, 125.91, 127.01, 127.45, 129.06, 129.43, 131.52, 132.66, 133.04, 141.01, 141.75, 148.43, 148.94, 152.96, 153.87, 166.51, 167.34, 181.07, 182.69.
HRMS (ESI), m/z: (M+H) found 241.0979, C_14_H_13_N_2_O_2_ requires 241.0977; (M+Na) found 263.0797, C_14_H_12_N_2_O_2_Na requires 263.0796.
(*E*,*Z*)-3-(4-Hydroxybenzylidene)-2-oxindole **(*E*)-14**
From 0.100 g (0.8 mmol) of 2-oxindole, 0.092 g (0.8 mmol) of 4-hydroxybenzaldehyde and 60 µL (0.7 mmol) of piperidine with ethanol as solvent the yellow powder (0.136 g, 76%) was obtained as a mixture of two isomers. According to ^1^H NMR *E*/*Z* stereoisomeric ratio is 19:1, respectively [29].
^1^H NMR (400.13 MHz, DMSO-*d_6_*, ppm): 6.82–6.93 (m, 4H), 7.19 (t, *J* = 7.6 Hz, 1H, H_ind_), 7.53 (s, 1H, CH=), 7.61 (d, *J* = 8.5 Hz, 2H, H_Ar_), 7.69 (d, *J* = 7.6 Hz, 1H, H_4_), 10.14 (br.s, 1H, OH), 10.52 (br. s, 1H, NH).
(*E*,*Z*)-3-(4-Hydroxybenzylidene)-2-oxindole **(*E*,*Z*)-14**
From 0.100 g (0.8 mmol) of 2-oxindole, 0.092 g (0.8 mmol) of 4-hydroxybenzaldehyde and 60 µL (0.7 mmol) of piperidine in 1,4-dioxane or ethyl acetate as solvent the yellow powder was obtained as a mixture of two isomers. Yields: 0.150 g, 84% (1,4-dioxane); 0.109 g, 61% (ethyl acetate). According to ^1^H NMR *E*/*Z* stereoisomeric ratio is 1.75:1, respectively. ^1^H NMR (400.13 MHz, DMSO-*d_6_*, ppm) for *E* isomer was identical to **14.**
Selected peaks of *Z* isomer, ^1^H NMR (400.13 MHz, DMSO-*d_6_*, ppm): 6.79 (d, *J* = 7.6 Hz, 1H, H_7_), 6.94 (td, *J*_1_ = 7.5 Hz, *J*_2_ = 0.6 Hz, 1H, H_ind_), 7.13 (td, *J*_1_ = 7.7 Hz, *J*_2_ = 0.6 Hz, 1H, H_ind_), 7.64 (s, 1H, CH=), 8.39 (d, *J* = 8.7 Hz, 2H, H_2′_, H_6′_)
(*E*,*Z*)-3-((5-Ethoxycarbonyl-1H-pyrazol-4-yl)methylidene)-2-oxindole **(*E*,*Z*)-39**
From 0.100 g (0.8 mmol) of 2-oxindole, 0.134 g (0.8 mmol) of ethyl 4-formyl-*1H*-pyrazole-5-carboxylate and 60 µL (0.7 mmol) of piperidine with ethanol, 1,4-dioxane or ethyl acetate as solvent the yellow powder was obtained as a mixture of two isomers. Yields: 0.136 g, 76% (ethanol); 0.154 g, 86% (1,4-dioxane); 0.120 g, 67% (ethyl acetate). According to ^1^H NMR major to minor stereoisomer ratio is 1.6:1 in all solvents [29].
Major isomer ^1^H NMR (400.13 MHz, DMSO-*d_6_*, ppm): 1.35 (t, *J* = 7.1 Hz, 3H, CH_3_), 4.33–4.40 (m, 2H, CH_2_), 6.5 (m, 1H, H_7_), 6.99 (td, *J*_1_ = 7.5 Hz, *J*_2_ = 0.7 Hz, 1H, H_ind_), 7.17–7.24 (m, 1H, H_ind_), 7.47 (d, *J* = 7.5 Hz, 1H, H_4_), 8.23 (s, 1H, CH=), 9,28 (br.s, 1H, H_pyrazol_), 10.64 (s., 1H, NH_ind_).
Selected peaks of minor isomer ^1^H NMR (400.13 MHz, DMSO-*d_6_*, ppm): 1.27 (t, *J* = 7.1 Hz, 3H, CH_3_), 4.25–4.33 (m, 2H, CH_2_), 6.90 (td, *J*_1_ = 7.6 Hz, *J*_2_ = 0.9 Hz, 1H), 7.62 (d, *J* = 7.6 Hz, 1H, H_4_), 7.82 (s, 1H, CH=), 8.42 (br.s, 1H, H_pyrazol_), 10.54 (s, 1H, NH_ind_).
3-((3,5-Dimethyl-1-phenyl-1H-pyrazol-4-yl)methylidene)-2-oxindole **44**
From 0.100 g (0.8 mmol) of 2-oxindole, 0.160 g (0.8 mmol) of 3,5-dimethyl-1-phenyl-*1H*-pyrazole-4-carbaldehyde and 60 µL (0.7 mmol) of piperidine with ethanol, 1,4-dioxane or ethyl acetate as solvent yellow powder was obtained as single isomer. Yields: 0.159 g, 63% (ethanol); 0.157 g, 62% (1,4-dioxane); incomplete conversion of started oxindole (76% conversion) in ethyl acetate. According to ^1^H NMR a single isomer was obtained in all solvents. [29]
^1^H NMR (400.13 MHz, DMSO-*d_6_*, ppm): 2.15–2.22 (m, 6H, CH_3_), 6.87 (d, *J* = 7.6 Hz, 1H, H_7_), 6.92 (td. *J*_1_ = 7.6 Hz, *J*_2_ = 0.7 Hz, 1H, H_ind_), 7.07 (d, *J* = 7.7 Hz, 1H, H_4_), 7.20 (td, *J*_1_ = 7.6 Hz, *J*_2_ = 0.8 Hz, 1H, H_ind_), 7.4–7.48 (m, 2H, H_ar,_ CH=), 7.53 (t, *J* = 7.5 Hz, 2H, H_ar_), 7.57–7.62 (m, 2H, H_ar_), 10.60 (br.s, 1H).

#### 3.1.3. General Procedure for Synthesis of 3-arylidene-2-oxindoles using MW Activation

2-Oxindole (1 eq.) and corresponding carboxaldehyde (1 eq.) were dissolved in 2–3 mL of 1,4-dioxane and 60 µL of piperidine was added. Reaction mixture was subjected to microwave irradiation (260W) for 11.5 min via a repeated process of 0.5–1 min of MW activation followed by a 1–2 min cooldown period. The reaction was carried out until the parent oxindole was no longer detected by TLC. The reaction mixture was cooled down to room temperature. 

If the precipitate formed, it was filtered off, washed with ethyl acetate and diethyl ether and dried. 

If no precipitate appeared, the solution was concentrated in vacuum and the resulting product was washed with ethyl acetate and diethyl ether and dried. The following compounds were obtained according to this procedure:

(*E*,*Z*)-3-(2-Pyridinylmethylidene)-2-oxindole **(*E*,*Z*)-1**
From 0.100 g (0.8 mmol) of 2-oxindole, 0.08 mL (0.8 mmol) of 2-pyridinecarboxaldehyde and 60 µL (0.67 mmol) of piperidine the brown oil (0.149 g, 83%) was obtained as a mixture of isomers. According to ^1^H NMR E:Z stereoisomer ratio is 3.7:1, respectively. ^1^H NMR signals were identical to described in Section 3.1.2. for 1.
(*E*,*Z*)-3-(4-Hydroxybenzylidene)-2-oxindole **(*E*,*Z*)-14**
From 0.100 g (0.8 mmol) of 2-oxindole, 0.092 g (0.8 mmol) of 4-hydroxybenzaldehyde and 60 µL (0.67 mmol) of piperidine the yellow powder (0.159 g, 89%) was obtained as a mixture of two isomers. According to ^1^H NMR *E*/*Z* stereoisomer ratio is 1.75:1, respectively. ^1^H NMR signals were identical to described in Section 3.1.2. for **14**.
(*E*,*Z*)-3-((5-Ethoxycarbonyl-1H-pyrazol-4-yl)methylidene)-2-oxindole **(*E*,*Z*)-39**
From 0.100 g (0.8 mmol) of 2-oxindole, 0.134 g (0.8 mmol) of ethyl 4-formyl-*1H*-pyrazole-5-carboxylate and 60 µL (0.67 mmol) of piperidine the yellow powder (0.089 g, 50%) was obtained as a mixture of two isomers. According to NMR ^1^H major to minor stereoisomer ratio is 1.6:1. ^1^H NMR signals were identical to described in Section 3.1.2. for ***(E,Z*)-39**.
3-((3,5-Dimethyl-1-phenyl-1H-pyrazol-4-yl)methylidene)-2-oxindole **44**
From 0.100 g (0.8 mmol) of 2-oxindole, 0.160 g (0.8 mmol) of 3,5-dimethyl-1-phenyl-*1H*-pyrazole-4-carbaldehyde and 60 µL (0.67 mmol) of piperidine the yellow powder was obtained as single isomer ^1^H NMR signals were identical to described in Section 3.1.2 for **44**. According to ^1^H NMR conversion is 68%.

#### 3.1.4. Synthesis of 3-(pyridin-2-ylmethyl)-5-amino-2-oxindole 47

To the suspension of 0.146 g (0.6 mmol) 3-(2-pyridinylmethylidene)-5-nitro-2-oxindole and 0.350 g (5.6 mmol) zinc powder in 5 mL of MeOH the 0.6 mL of HCl conc. was rapidly added with vigorous stirring. After 30 min, the reaction was terminated by addition of NaHCO_3_ and pH was adjusted to 8. The reaction mixture was extracted with EtOAc, organic fraction was dried with Na_2_SO_4_ and the solvent was removed under reduced pressure. The compound (0.086 g, 65%) was obtained as brown powder.
^1^H NMR (400.13 MHz, DMSO-*d_6_*, ppm): 2.88–3.0 (m, 1H, CH_2_), 3.28–3.36 (m, 1H, CH_2_), 3.83–3.90 (m, 1H, CH), 4.51 (br.s, 2H, NH_2_), 5.98 (s, 1H, H_4_), 6.33 (dd, *J*_1_ = 7.6 Hz, *J*_2_ = 1.8 Hz, 1H, H_6_), 6.49 (d, *J* = 8.1 Hz, 1H, H_7_), 7.2–7.26 (m, 2H, H_py_), 7.65–7.72 (td, *J*_1_ = 7.7 Hz, *J*_2_ = 1.8 Hz, 1H, H_py_), 8.495 (d, *J* = 4.1 Hz, 1H, H_3′_), 9.99 (br. s, 1H, NH).
^13^C NMR (100.6 MHz, DMSO-*d_6_*, ppm): 38.15, 45.04, 109.46, 111.31, 112.63, 121.76, 123.60, 130.41, 132.34, 136.42, 143.20, 149.03, 158.41, 178.07.
HRMS (ESI), *m*/*z*: (M+H) found 240.1128, C_14_H_14_N_3_O requires 240.1137, (M+Na) found 262.0939, C_14_H_13_N_3_ONa requires 262.0956.

### 3.2. Biology

#### 3.2.1. NQO2 Assay

The activity of recombinant human NQO2 (Sigma #Q0380, St. Louis, MA, USA) was evaluated kinetically using menadione and *N*-benzyl-dihydronicotinamide (BNAH) as the substrate and co-substrate, respectively. All reagents and test compounds were dissolved in 50 mM Hepes-KOH (pH 7.4) containing 1 mM of *β-*octyl-*D*-glucopyranoside, 0.1 mg/mL BSA and 1 μM FAD. In a 96-well black flat-bottom plate, 50 μL of the test compounds were introduced at a final concentration of 10 μM for primary screening, and a range of final concentrations from 10 nM to 100 μM was used to determine IC_50_ values. Quercetin was used as a positive control. The solutions of the test compounds were pre-incubated for 5 min with 50 μL of human recombinant NQO2 (final concentration 42 ng/mL). Then, 25 μL of menadione was introduced at a final concentration of 100 μM. The reaction was started with 25 μL of 100 μM BNAH. The fluorescence of the co-substrate BNAH was followed at wavelengths of 370/440 nm at 37 °C using an Infinite M200 Pro microplate reader (Tecan, Grödig, Austria). The fluorescence data was fit according to a one-phase decay nonlinear regression to obtain slope values with Prism 8.0 (GraphPad Inc., San Diego, CA, USA). The activity in sample wells were normalized against negative control and enzyme-blank samples.

#### 3.2.2. Cell Culture

A549 lung carcinoma cells were purchased from the ATCC (CCL-185). The A549 cells were maintained in F12-K (Gibco, Paisley, UK) supplemented with 10% fetal bovine serum (FBS, Gibco, UK), penicillin (100 UI mL^−1^), streptomycin (100 µg mL^−1^) and GlutaMax (2 mM, Gibco, UK). All cells line were cultured under a humidified atmosphere of 95% air/5% CO_2_ at 37 °C. Subconfluent monolayers, in the log growth phase, were harvested by a brief treatment with TrypLE Express solution (Gibco, UK) in phosphate-buffered saline (PBS, Capricorn Scientific, Ebsdorfergrund, Germany) and washed three times in serum-free PBS. The number of viable cells was determined by trypan blue exclusion.

#### 3.2.3. Antiproliferative Assay

The effects of the synthesized compounds on cell viability were determined using the MTT colorimetric test. All examined cells were diluted with the growth medium to 3.5 × 10^4^ cells per mL and the aliquots (7 × 10^3^ cells per 200 μL) were placed in individual wells in 96-multiplates (Eppendorf, Germany) and incubated for 24 h. The next day the cells were treated with the synthesized compounds separately at various concentrations for the determination of CC50 and incubated for 72 h at 37 °C in a 5% CO_2_ atmosphere. After incubation, the cells were then treated with 40 μL MTT solution (3-(4,5-dimethylthiazol-2-yl)-2,5-diphenyltetrazolium bromide, 5 mg mL^−1^ in PBS) and incubated for 4 h. After an additional 4 h incubation, the medium with MTT was removed and DMSO (150 μL) was added to dissolve the formazan crystals. The plates were shaken for 10 min. The optical density of each well was determined at 560 nm using a microplate reader GloMax Multi+ (Promega, Madison, WI, USA). Each of the tested compounds was evaluated for cytotoxicity in three separate experiments.

### 3.3. In Silico Studies

The in silico studies were carried out according to the standard algorithms of the Schrodinger software package and our previous work **[38].**

## 4. Conclusions

The synthesis and study of 3-arylidene-2-oxindoles as novel NRH:quinone oxidoreductase 2 inhibitors was performed. It was shown that the *E*-isomer was the predominant product in the synthesis of 3-benzylidene and 3-(pyridylmethylidene) derivatives, while for pyrazole-4-carbaldehyde derivatives, the *Z*-isomer sometimes predominated. An NMR criterion for determining the (*E*/*Z*)-configuration of 3-(pyridin-2-ylmethylidene)-2-oxindoles was proposed. It was shown that for *E*-isomers, the signals of protons in the 4th position of the oxindole ring are characteristic in the 1H NMR spectra and lie in the region of 8.9–10 ppm. The molecular modeling of binding to the active site of NQO2 demonstrated that both the *E-* and *Z*-isomers are capable of π–π stacking with the FAD cofactor and are located in the active site of the enzyme similar to MCA-NAT (a selective inhibitor of NQO2). The presence of hydroxy groups in the arylidene moiety and the introduction of a methyl group to the oxindole nitrogen led to an increase in binding affinity. The most active compounds **15**, **17**, **18**, **24**, and **39** inhibited NQO2 with IC_50_ values of 0.37–0.62 µM. No correlation was observed between the enzymatic inhibition activity and the cytotoxicity of 2-oxindole derivatives, so further investigation is required.

## Figures and Tables

**Table 1:** Preferred configurations of 4′-substituted benzylidene-2-oxindoles and corresponding characteristic chemical shifts.

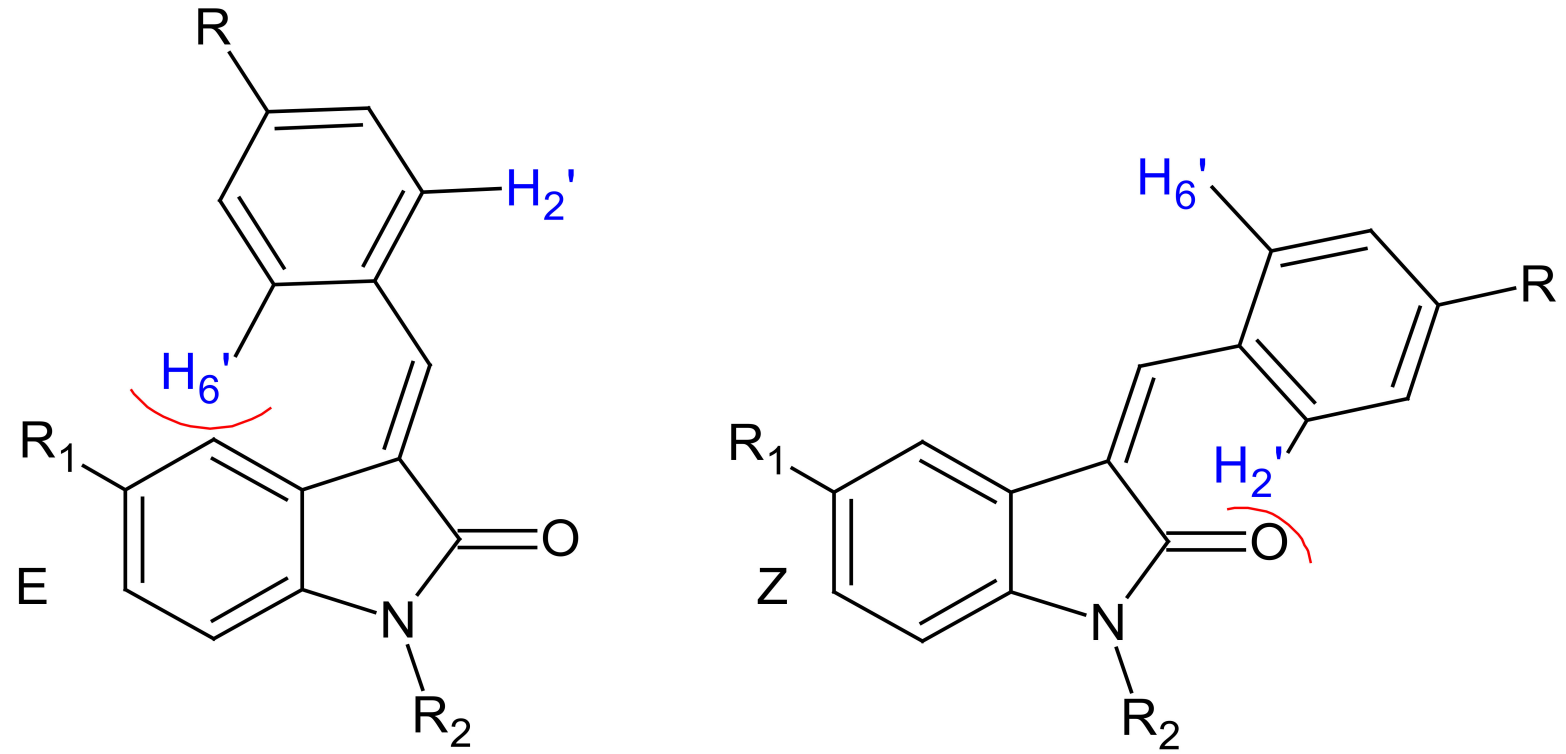						
№	R	R_1_	R_2_	Chemical Shift of H_2′_ H_6′_ (ppm)		E:Z Ratio
				E	Z	
14	-OH	H	H	7.47	8.24	19:1
15	-OH	H	Me	7.48	-	1:0
16	-OH	NHC(O)CH_3_	H	7.60	8.38	2.6:1
17	-OH	NHC(O)OCH_3_	H	7.63	8.41	25:1
18	-OH	NH(2-furoyl)	H	7.66	8.40	2:1
19	-OH	NHBz	H	7.67	8.42	2.3:1
20	-OMe	H	H	7.70	8.47	5:1
21	-OMe	NHBz	H	7.76	8.51	2:1
22	-OEt	H	H	7.69	-	1:0
28	-NO_2_	H	H	7.94	8.27	10:1
29	-NO_2_	NHC(O)OCH_3_	H	7.93	8.26	3:1
30	-NO_2_	NHBz	H	7.85	8.25	2:1
31	-N(Me)_2_	H	H	7.57–7.67 ^1^	8.44	1:1
32	-Br	Br	H	7.73	8.31	1.6:1
33	-Br	NHC(O)OCH_3_	H	7.67	8.31	12.5:1
34	-F	H	H	7.73–7.75 ^1^	8.45–8.50 ^1^	2:1

^1^ Multiplet signal.

**Table 2:** Preferred configuration of 3-(pyridin-2-ylmethylidene)-2-oxindoles and corresponding characteristic chemical shifts.

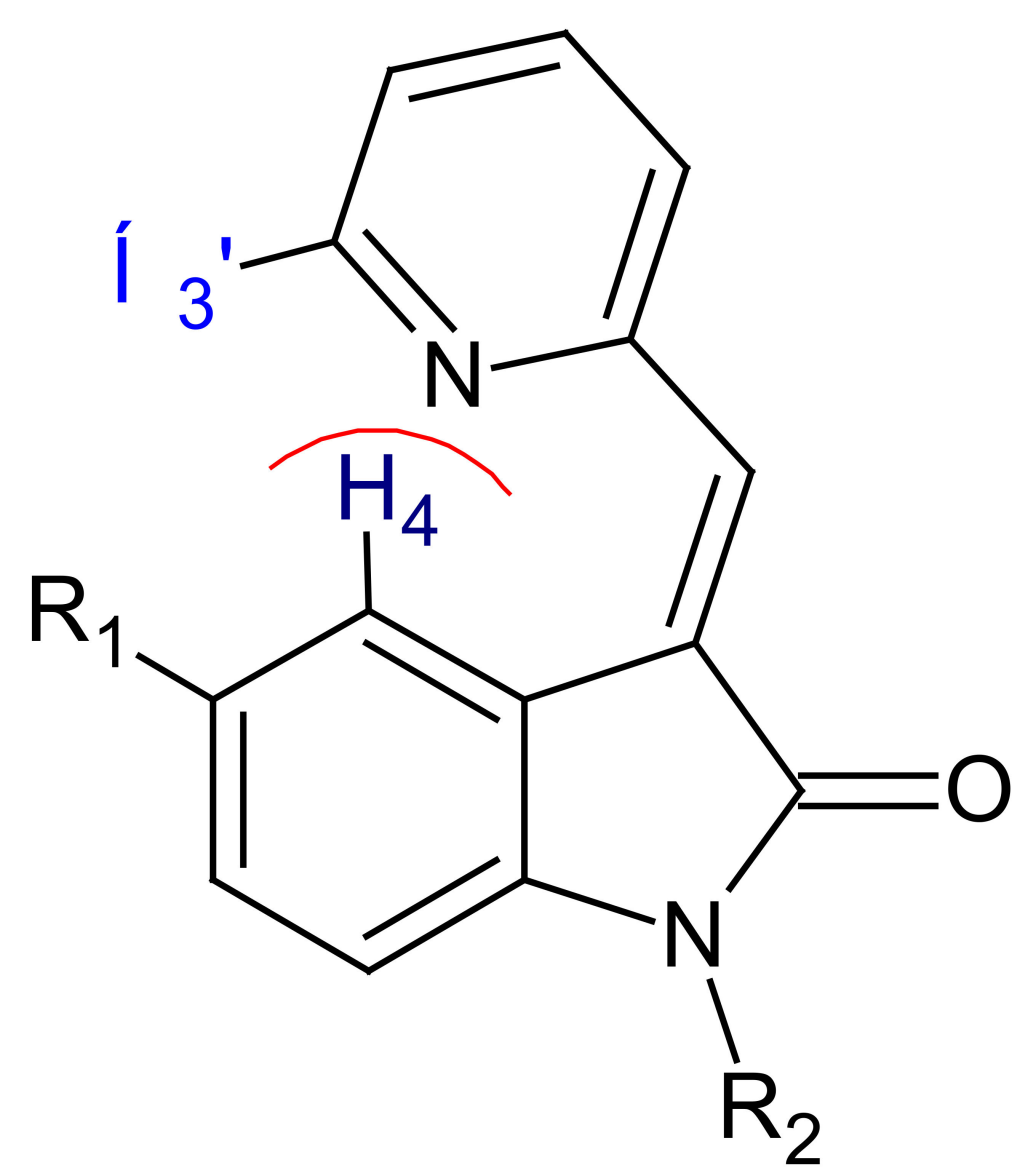					
№	R_1_	R_2_	*E*-Isomer		E:Z Ratio
			**Chemical Shift of H_4_ (ppm)**	**Chemical Shift of H_3′_ (ppm)**	
**1**	H	H	8.98	8.40	1:0
**2**	H	CH_3_	8.85–8.91 ^1^		1:0
**3**	Br	H	9.27	8.87	1:0
**4**	NO_2_	H	10.11	8.85	1:0
**5** ^2^	AcNH	H	9.31	8.89	7.7:1
**6** ^2^	BzNH	H	9.46	8.88	5:1

^1^ Multiplet signal; ^2^ The H_4_ signals of Z-isomer were undistinguishable.

**Table 3:** Influence of the reaction conditions on the ratio of isomers.

Activation Method		Thermal Activation (reflux)			MW
**Aldehyde**	Solvent	Ethanol	1,4-dioxane	Ethyl Acetate	1,4-dioxane
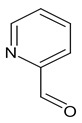	***E:Z*** **products ratio** **for compound 1**	1:0	**1a**, then 1.3:1 ^1,2^	**1a** as product ^1^	3.7:1
	**Reaction time, min**	120	90	10	11.5
	**Yield, %**	63	81	49	83
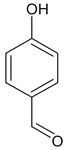	***E:Z*** **products ratio** **for compound 14**	19:1	2.3:1	2.3:1	2.3:1
	**Reaction time, min**	60	60	30	11.5
	**Yield, %**	76	84	61	89
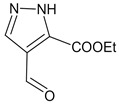	***E:Z*** **products ratio** **for compound 39**	1.6:1	1.6:1	1.6:1	1.6:1
	**Reaction time, min**	120	120	20	11.5
	**Yield, %**	61	86	67	50
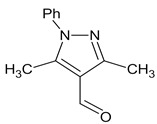	***E:Z*** **products ratio** **for compound 44**	1:0	1:0	1:0	1:0
	**Reaction time, min**	150	120	120	11.5
	**Yield, %**	63	62	Conversion 76%	Conversion 68%

^1^ See Scheme 2. ^2^ The intermediate **1a** was precipitated after 10 min of reaction, then the reaction proceeded and led to mixture of *E*/*Z*-isomers of **1.**

**Table 4:** Evaluation of enzyme inhibition activity and compound cytotoxicity against A549 cancer cell line.

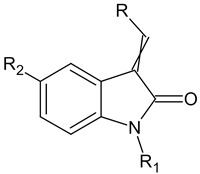				NQO2		A549, CC_50_ (μM)
№	R	R_1_	R_2_	Inhibition ^1^, %	IC_50_ ± SEM (μM)	
1	2-pyridyl	H	H	19.56	>>50	47.97 ± 4.42
2	2-pyridyl	Me	H	64.43	16.78 ± 0.09	- ^2^
3	2-pyridyl	H	Br	18.63	>>50	90.56 ± 1.50
4	2-pyridyl	H	NO_2_	1.85	>>50	n.a. ^3^
5	2-pyridyl	H	AcNH	46.35	41.13 ± 2.44	135.9
6	2-pyridyl	H	BzNH	43.13	n.d ^2^.	17.43 ± 1.80
7	3-pyridyl	H	H	59.17	n.d.	67.45 ± 7.58
8	3-pyridyl	H	Br	44.24	n.d.	22.30 ± 1.42
9	3-pyridyl	H	NO_2_	4.25	>>50	428.59 ± 23.97
10	4-pyridyl	H	H	27.29	>>50	80.27 ± 9.87
11	4-pyridyl	H	Br	41.92	n.d.	22.30 ± 1.42
12	4-pyridyl	H	NO_2_	19.23	>>50	n.a.
13	3-OH-C_6_H_4_	H	AcNH	89.07	0.64 ± 0.04	175.83 ± 4.76
14	4-OH-C_6_H_4_	H	H	31.68	>>50	80.85 ± 12.22
15	4-OH-C_6_H_4_	Me	H	98.87	0.62 ± 0.04	n.a.
16	4-OH-C_6_H_4_	H	AcNH	70.41	0.99 ± 0.03	n.a.
17	4-OH-C_6_H_4_	H	MeOC(O)NH	93.63	0.44 ± 0.02	44.43 ± 0.29
18	4-OH-C_6_H_4_	H	2-furoylNH	96.4	0.37 ± 0.02	146.25 ± 11.31
19	4-OH-C_6_H_4_	H	BzNH	62.89	n.d.	412.17 ± 3.98
20	4-OMe-C_6_H_4_	H	H	40.82	n.d.	54.76
21	4-OMe-C_6_H_4_	H	BzNH	30.89	>>50	42.33 ± 8.30
22	4-OEt-C_6_H_4_	H	H	25.41	>>50	-
23	3,4,5-triOMe-C_6_H_2_	H	H	85.71	1.6 ± 0.14	25.72
24	3,4,5-triOMe-C_6_H_2_	H	AcNH	92.78	0.37 ± 0.01	148.99 ± 2.63
25	3,4,5-triOMe-C_6_H_2_	H	2-furoylNH	90.98	0.66 ± 0.02	200.90 ± 22.65
26	3,4-diOMe-4-OH-C_6_H_2_	H	BzNH	40.47	n.d.	n.a.
27	4-NO_2_-C_6_H_4_	H	H	4.66	>>50	-
28	4-NO_2_-C_6_H_4_	H	MeOC(O)NH	74.0	n.d.	n.a.
29	4-NO_2_-C_6_H_4_	H	BzNH	21.79	>>50	-
30	4-NMe_2_-C_6_H_4_	H	H	27.30	>>50	n.a.
31	4-Br-C_6_H_4_	H	Br	68.63	n.d.	241.71 ± 3.19
32	4-Br-C_6_H_4_	H	MeOC(O)NH	95.46	0.61 ± 0.03	n.a.
33	4-F-C_6_H_4_	H	H	19.46	>>50	38.56 ± 10.45
34	2-furyl	H	H	59.91	n.d.	594.74 ± 25.37
35	2-furyl	H	Br	−15.2	>>50	449.07 ± 18.72
36	2-furyl	H	NO_2_	59.55	n.d.	328.55 ± 17.00
37	2-thienyl	H	H	38.1	n.d.	491.01 ± 19.85
38	2-thienyl	H	Br	29.9	>>50	426.53 ± 6.20
39	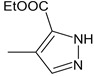	H	H	47.32	0.34 ± 0.02	231.3
40	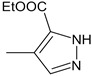	H	NO_2_	92.3	1.88 ± 0.08	-
41	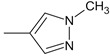	H	H	59.76	9.40 ± 0.12	-
42	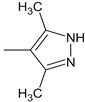	H	H	54.35	n.d.	-
43	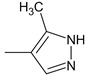	H	H	77.81	7.61 ± 0.19	218.9
44	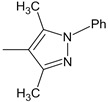	H	H	43.58	n.d.	n.a.
45	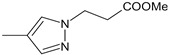	H	H	28.97	>>50	n.a.
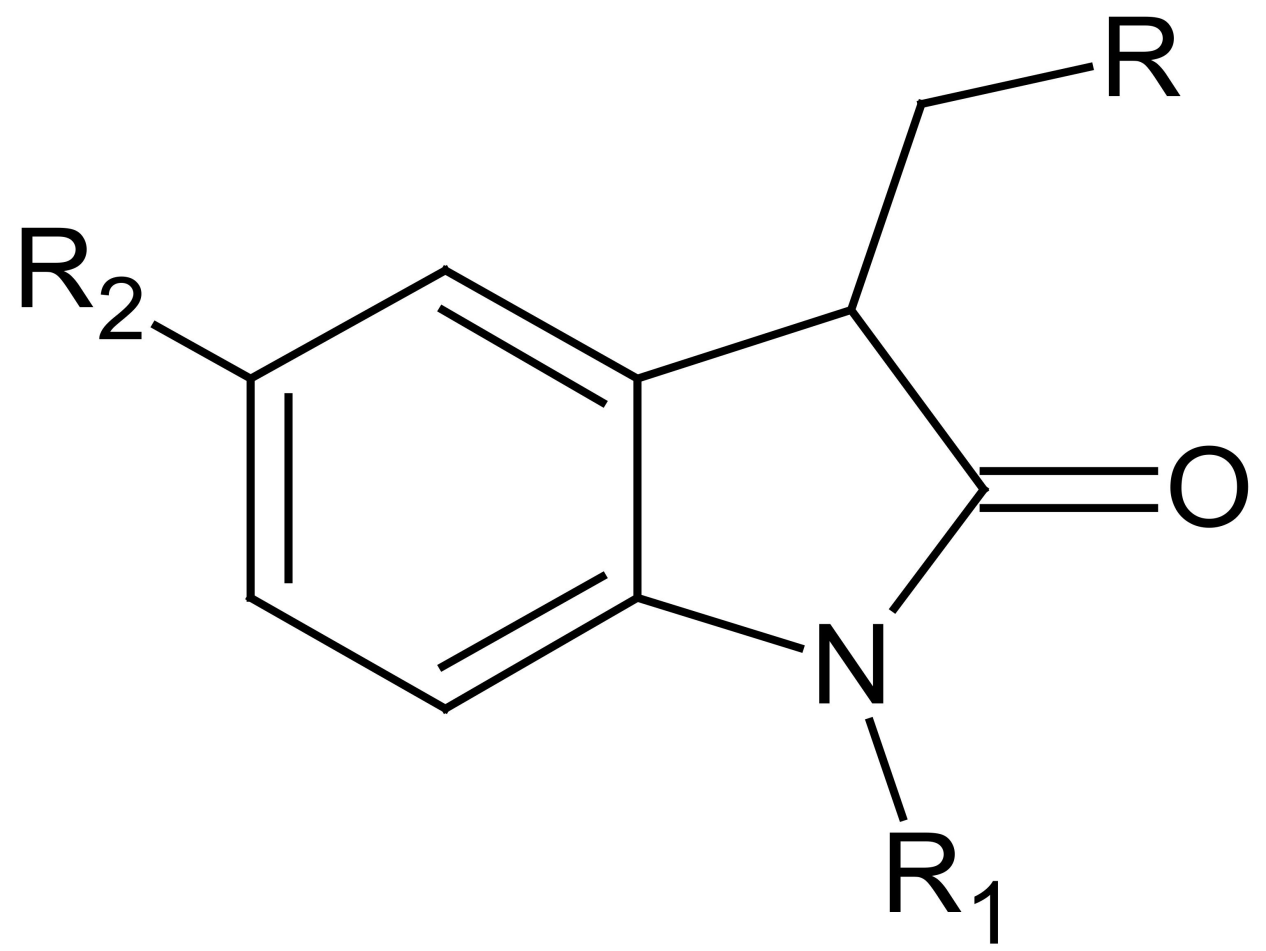						
**№**	**R**	**R_1_**	**R_2_**			
46	4-NH_2_-C_6_H_4_	H	H	19.05	>>50	-
47	2-pyridyl	H	NH_2_	26.93	>>50	n.a.
	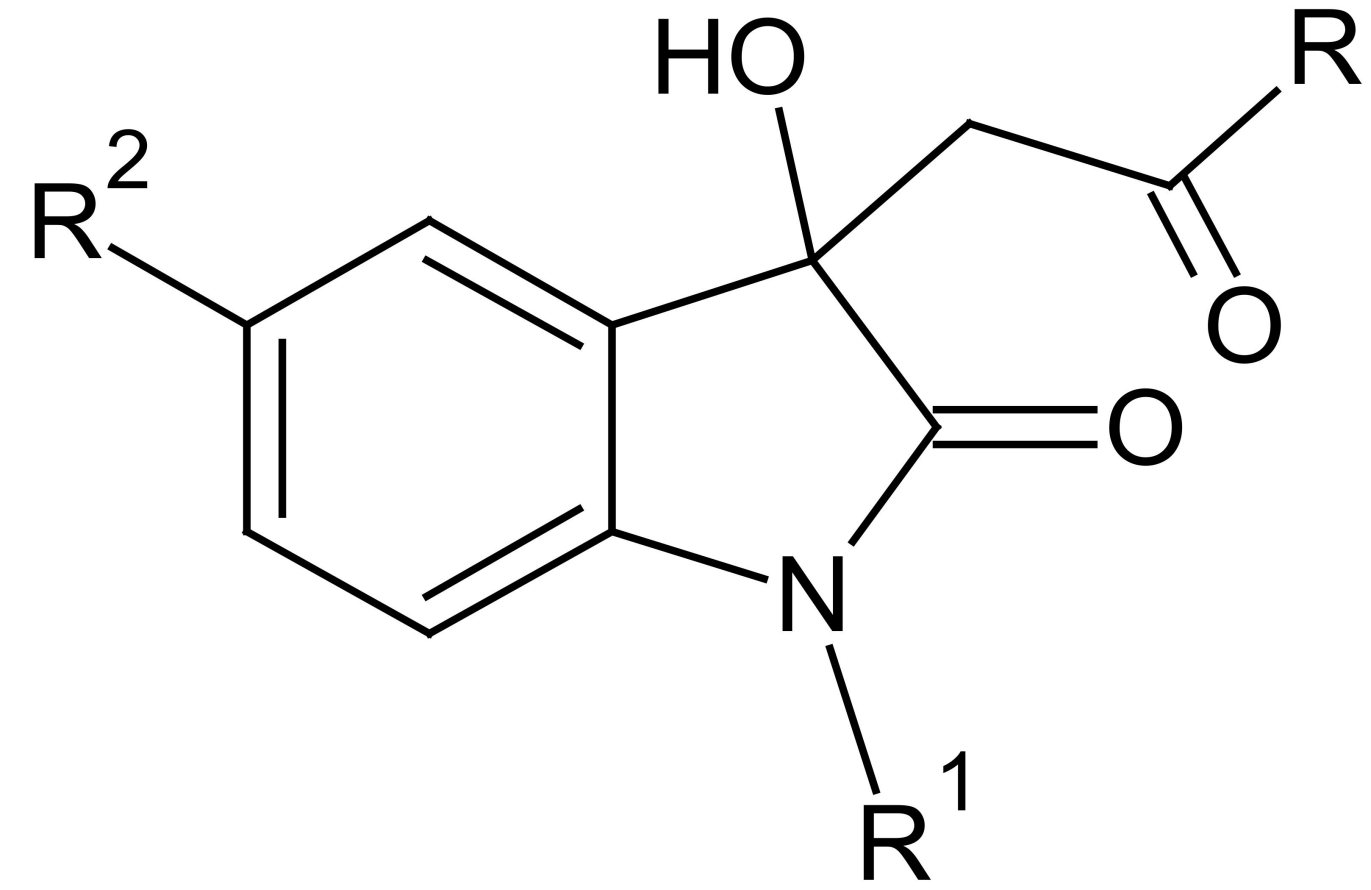					
**№**	**R**	**R_1_**	**R_2_**			
48	4-Me-C_6_H_4_	H	OMe	−50.28	>>50	n.a.
49	4-Me-C_6_H_4_	H	Br	−85.22	>>50	n.a.
50	4-MeO-C_6_H_4_	H	Br	78.82	n.d.	473.55 ± 18.21
51	4-Me-C_6_H_4_	H	2-furoyl-NH	25.63	>>50	n.a.
52	4-Me-C_6_H_4_	H	H	−92.90	>>50	170.63 ± 13.41
Melatonin					63.5 ± 2.7	
Quercetin				98	0.08 ± 0.02	

^1^ inhibition at 10 µM concentration; ^2^ not determined; ^3^ not active.

## Data Availability

The data presented in this study are available on request from the corresponding authors.

## Supplementary Materials

The following supporting information can be downloaded at: www.mdpi.com/article/10.3390/molecules28031174/s1, Figures S1–S20: 1H and 13C NMR spectra of synthesized compounds; Figures S21 and S22: concentration dependence of inhibition activity for the most active compounds and Michaelis–Menten kinetic study for compound **15**; Figure S23: docking simulation for compound **24**; Table S1: docking score for most active compounds **15**,**17**,**18**,**24**, and **39**.
