# Supplementary material for: 3-Arylidene-2-oxindoles as Potent NRH:Quinone Oxidoreductase 2 Inhibitors
Source: Molecules. 2023 Jan 25;28(3):1174. doi: 10.3390/molecules28031174 (PMC9920986; doi:10.3390/molecules28031174)
Supplement: Supplementary file 1 [file molecules-28-01174-s001.zip › molecules-2119986-supplementary.pdf]

# Supplementary Materials

# 3-Arylidene-2-oxindoles as potent NRH:quinone oxidoreductase 2 inhibitors

**Natalia A. Lozinskaya[1*], Elena N. Bezsonova[1], Meriam Dubar[1], Daria D. Melekhina[1], Daniil R. Bazanov[1], Alexander S. Bunev[2], Olga B. Grigor'eva[2], Vladlen G. Klochkov[3], Elena V. Sokolova[3], Denis A. Babkov[3], Alexander A. Spasov[3], and Sergey E. Sosonyuk*[1]**

[1] Department of Chemistry, Lomonosov Moscow State University, Moscow 119991, Russia
[2] Medicinal Chemistry Center, Togliatti State University, Togliatti, Russian Federation

[3] Department of Pharmacology & Bioinformatics, Volgograd State Medical University, Volgograd 400131, Russia
* Correspondence: natalylozinskaya@mail.ru (N.A.L.); umpolung@yandex.ru (S.E.S.)

# Contents


NMR SPECTRA OF SYNTHESIZED COMPOUNDS ........ 3

**S1. 1H NMR spectrum of (*E,Z*)-3-(2-pyridinylmethylidene)-5-acetamido-2-oxindole 5 ........ 4**

**S2. 13C NMR spectrum of (*E,Z*)-3-(2-pyridinylmethylidene)-5-acetamido-2-oxindole 5 ........ 5**

**S3. 1H NMR spectrum of (*E,Z*)-3-(2-pyridinylmethylidene)-5-benzoylamino-2-oxindole 6 ........ 6**

**S4. 13C NMR spectrum of (*E,Z*)-3-(2-pyridinylmethylidene)-5-benzoylamino-2-oxindole 6 ........ 7**

**S5. 1H NMR spectrum of *(E,Z*)-3-(4-hydroxybenzylidene)-5-acetamido-2-oxindole 16 ........ 8**

**S6. 13C NMR spectrum of *(E,Z*)-3-(4-hydroxybenzylidene)-5-acetamido-2-oxindole 16 ........ 9**

**S7. 1H NMR spectrum of *(E,Z*)-3-(4-methoxybenzylidene)-5-benzoylamino-2-oxindole 21 ........ 10**

**S8. 13C NMR spectrum of *(E,Z*)-3-(4-methoxybenzylidene)-5-benzoylamino-2-oxindole 21 ........ 11**

**S9. 1H NMR spectrum of (*E*)-3-(4-ethoxybenzylidene)-2-oxindole 22 ........ 12**

**S10. 1H NMR spectrum of (*E,Z*)-3-(3,4,5-trimethoxybenzylidene)-2-oxindole 23 ........ 13**

**S11. 1H NMR (*E,Z*)-3-(3,5-dimethoxy-4-hydroxybenzylidene)-5-benzoylamino-2-oxindole 26 ........ 14**

**S12. 13C NMR spectrum of (*E,Z*)-3-(3,5-dimethoxy-4-hydroxybenzylidene)-5-benzoylamino-2-oxindole 26 ........ 15**

**S13. 1H NMR spectrum of (*E,Z*)-3-(4-dimethylaminobenzylidene)-2-oxindole 30 ........ 16**

**S14. 1H NMR spectrum of (*E,Z*)-3-(4-fluorobenzylidene)-2-oxindole 33 ........ 17**

**S15. 1H NMR spectrum of (*E,Z*)-3-(1-[2-(methoxycarbonyl)ethyl]-*1H*-pyrazol-4-ylmethylidene)-2-oxindole 45 ... 18**

**S16. 13C NMR spectrum of (*E,Z*)-3-(1-[2-(methoxycarbonyl)ethyl]-*1H*-pyrazol-4-ylmethylidene)-2-oxindole 45 .. 19**

**S17. 1H NMR spectrum of 3-(hydroxy(pyridin-2-yl)methyl)-2-oxindole 1a ........ 20**

**S18. 13C NMR spectrum of 3-(hydroxy(pyridin-2-yl)methyl)-2-oxindole 1a ........ 21**

**S19. 1H NMR spectrum of 3-pyridin-2-ylmethyl-5-amino-2-oxindole 47 ........ 22**

**S20. 13C NMR spectrum of 3-pyridin-2-ylmethyl-5-amino-2-oxindole 47 ........ 23**

BIOLOGICAL DATA ........ 24

**S21. Concentration dependence of NQO2 inhibition by some active compounds ........ 25**

**S22. Michaelis-Menten kinetic study for compound 15 ........ 26**

MOLECULAR MODELING ........ 27

**Figure S23. Proposed binding models of MCA-NAT and 24 to active site of NQO2 (blue) with FAD (green). A: binding pose of MCA-NAT (purple) and E (yellow) isomer of 24. B: the first pose of E (yellow) and the third one of Z (red) of 24. C: interaction map for the E isomer of 24. D: interaction map for the Z isomer of 24. ........ 27**

**Figure S24 the first pose of E (yellow) and the first one of Z (red) of 24 ........ 28**

**Table S1. Docking score for E and Z isomers of selected compounds ........................................................... 28**

# NMR spectra of synthesized compounds

S1. 1H NMR spectrum of (*E*,*Z*)-3-(2-pyridinylmethylidene)-5-acetamido-2-oxindole **5**

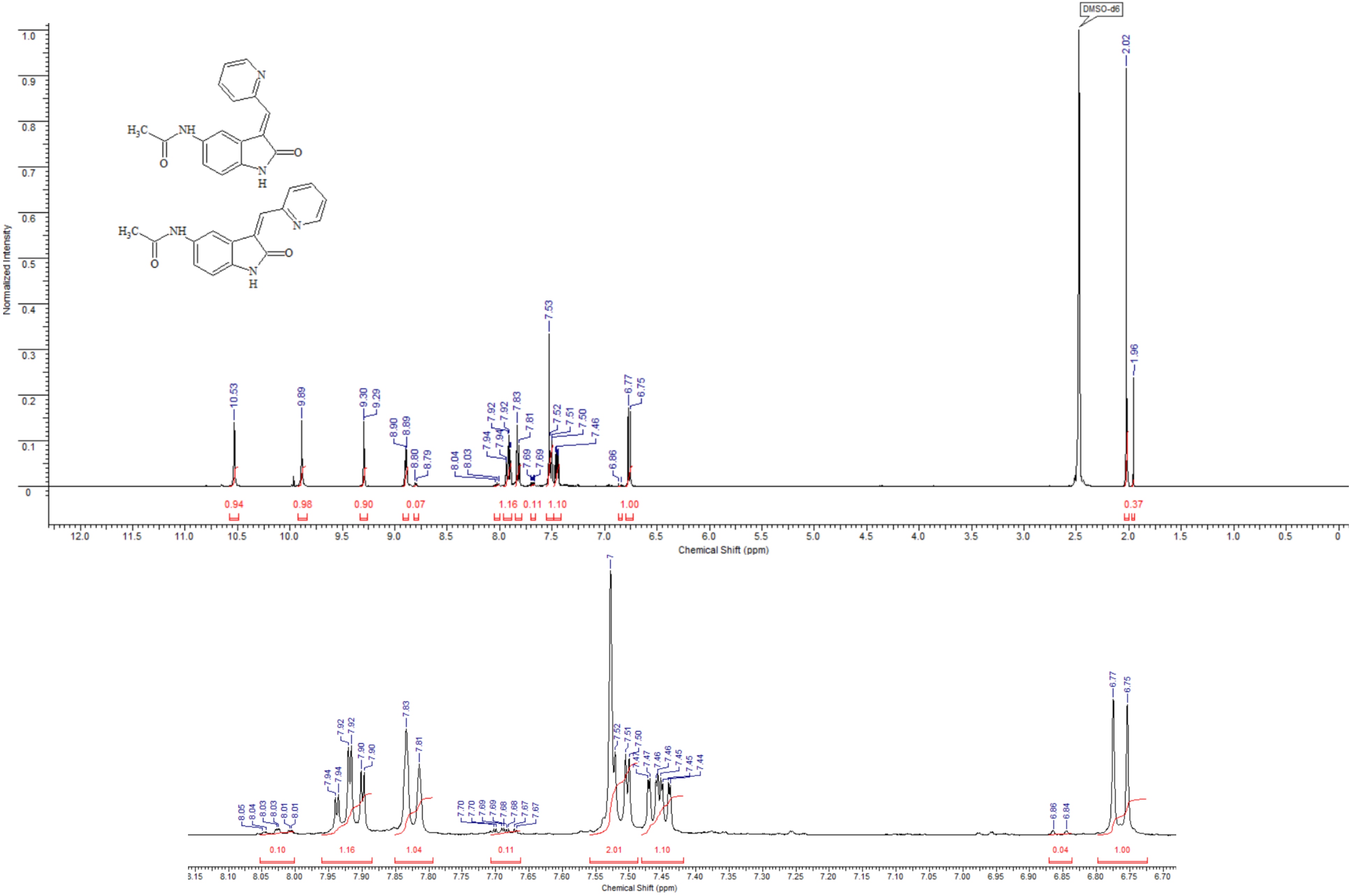

S2. 13C NMR spectrum of (*E*,*Z*)-3-(2-pyridinylmethylidene)-5-acetamido-2-oxindole **5**

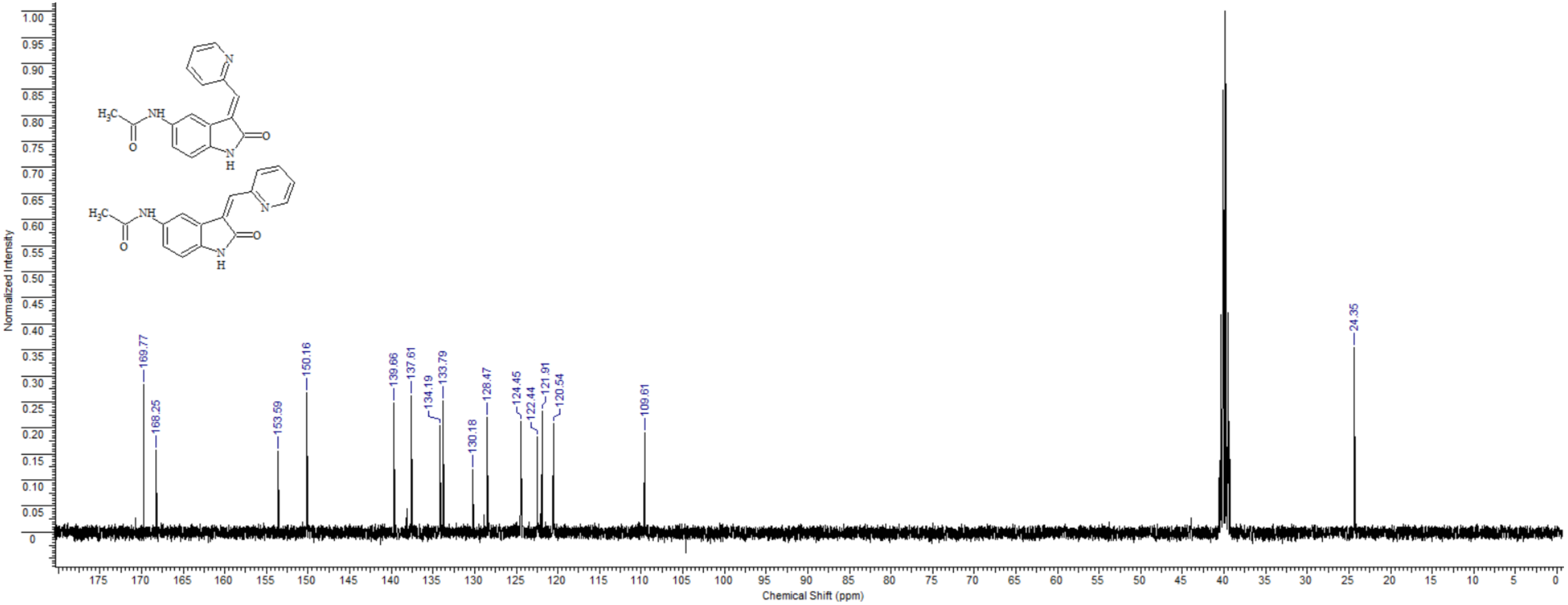

S3. 1H NMR spectrum of (*E*,*Z*)-3-(2-pyridinylmethylidene)-5-benzoylamino-2-oxindole **6**

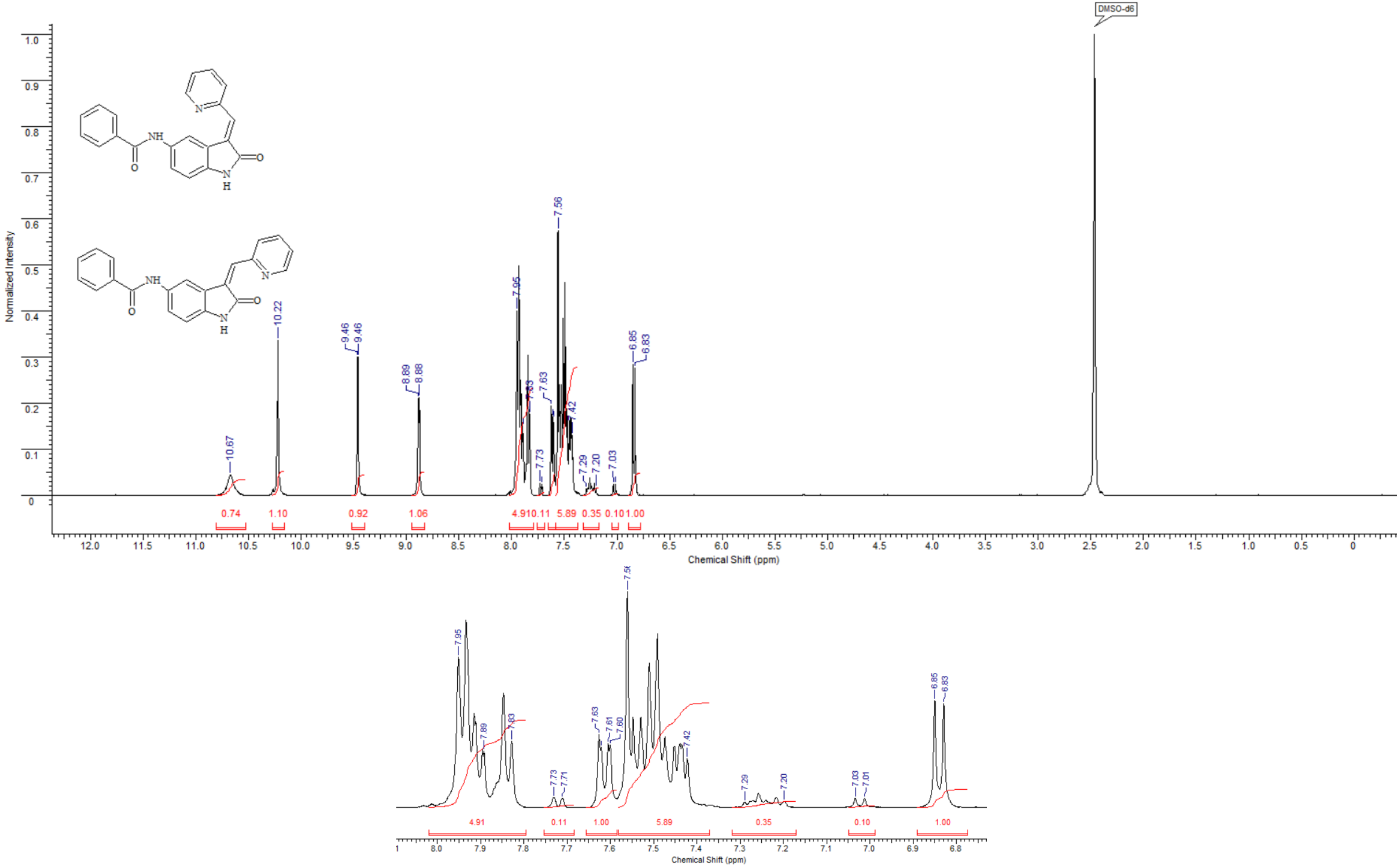

S4. 13C NMR spectrum of (*E*,*Z*)-3-(2-pyridinylmethylidene)-5-benzoylamino-2-oxindole **6**

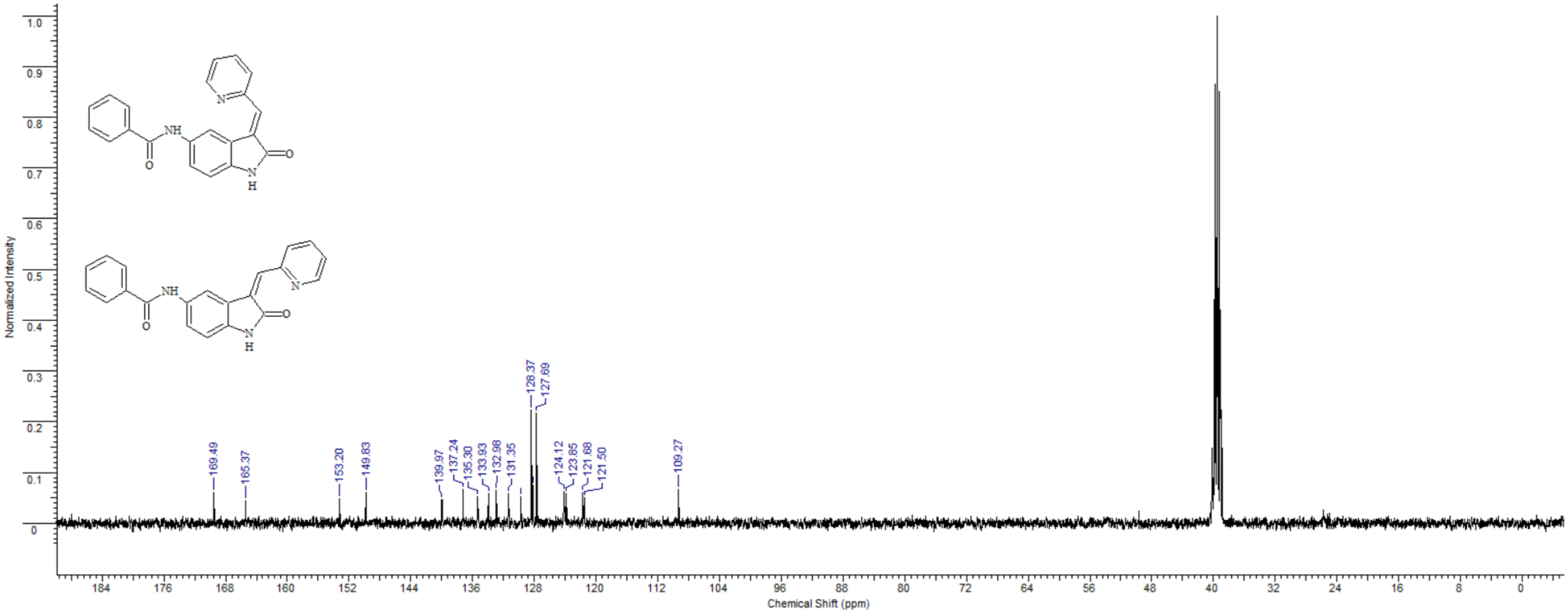

S5. 1H NMR spectrum of (*E*,*Z*)-3-(4-hydroxybenzylidene)-5-acetamido-2-oxindole **16**

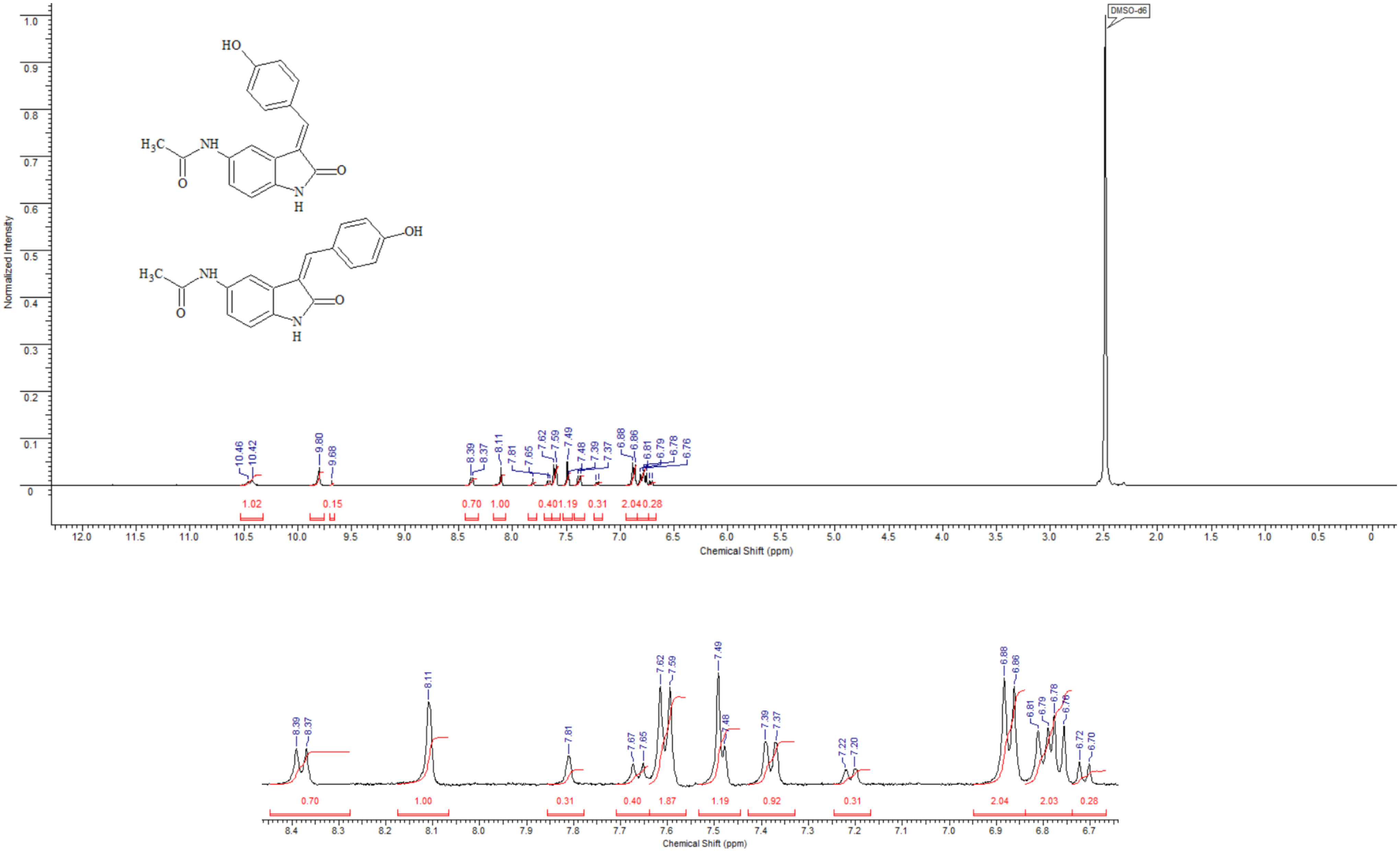

S6. 13C NMR spectrum of *(E,Z)*-3-(4-hydroxybenzylidene)-5-acetamido-2-oxindole **16**

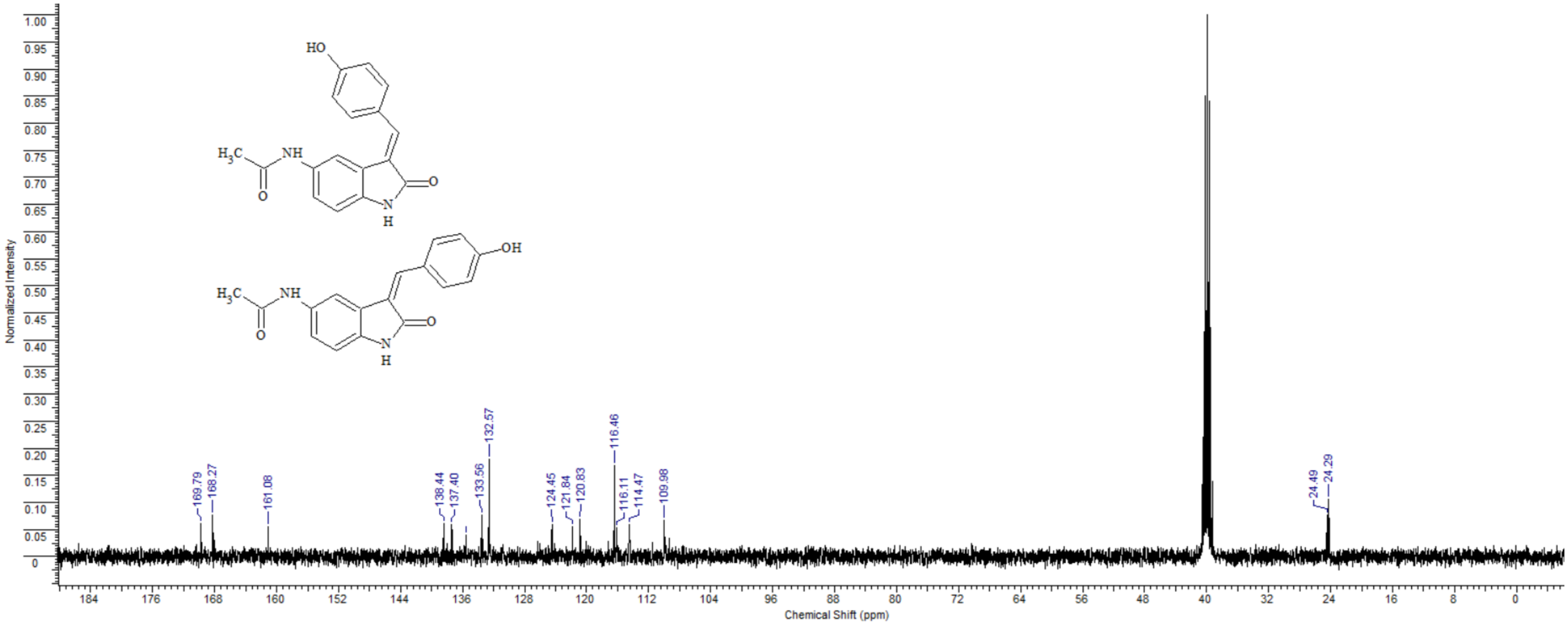

S7. 1H NMR spectrum of *(E,Z)*-3-(4-methoxybenzylidene)-5-benzoylamino-2-oxindole **21**

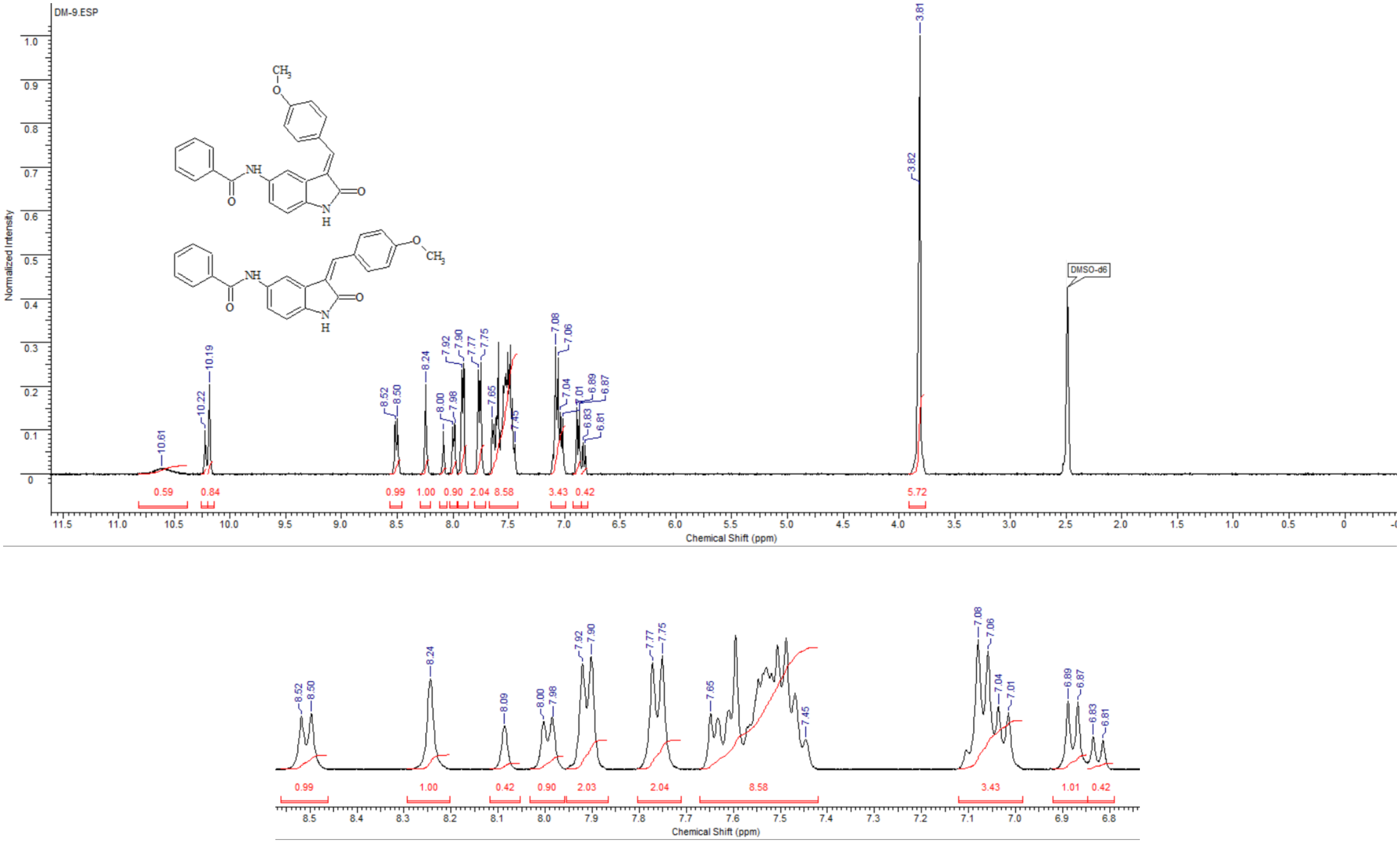

S8. 13C NMR spectrum of (*E*,*Z*)-3-(4-methoxybenzylidene)-5-benzoylamino-2-oxindole **21**

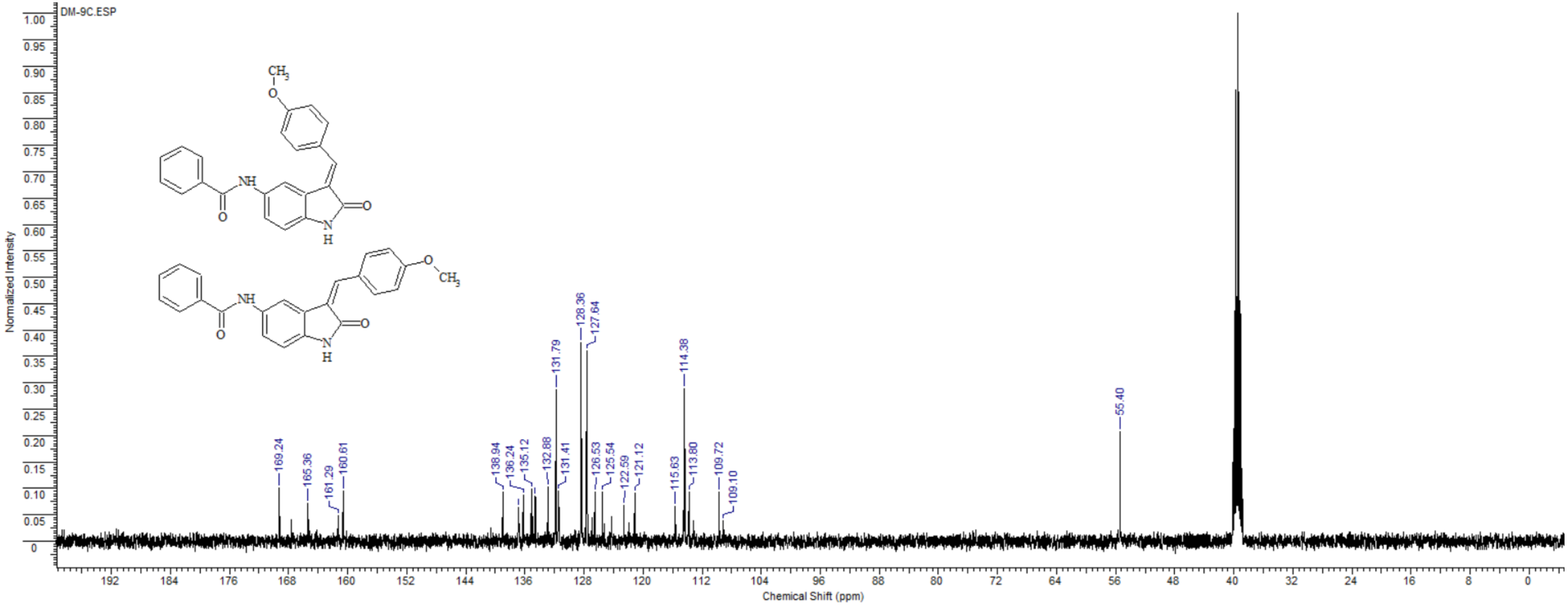

S9. 1H NMR spectrum of (*E*)-3-(4-ethoxybenzylidene)-2-oxindole **22**

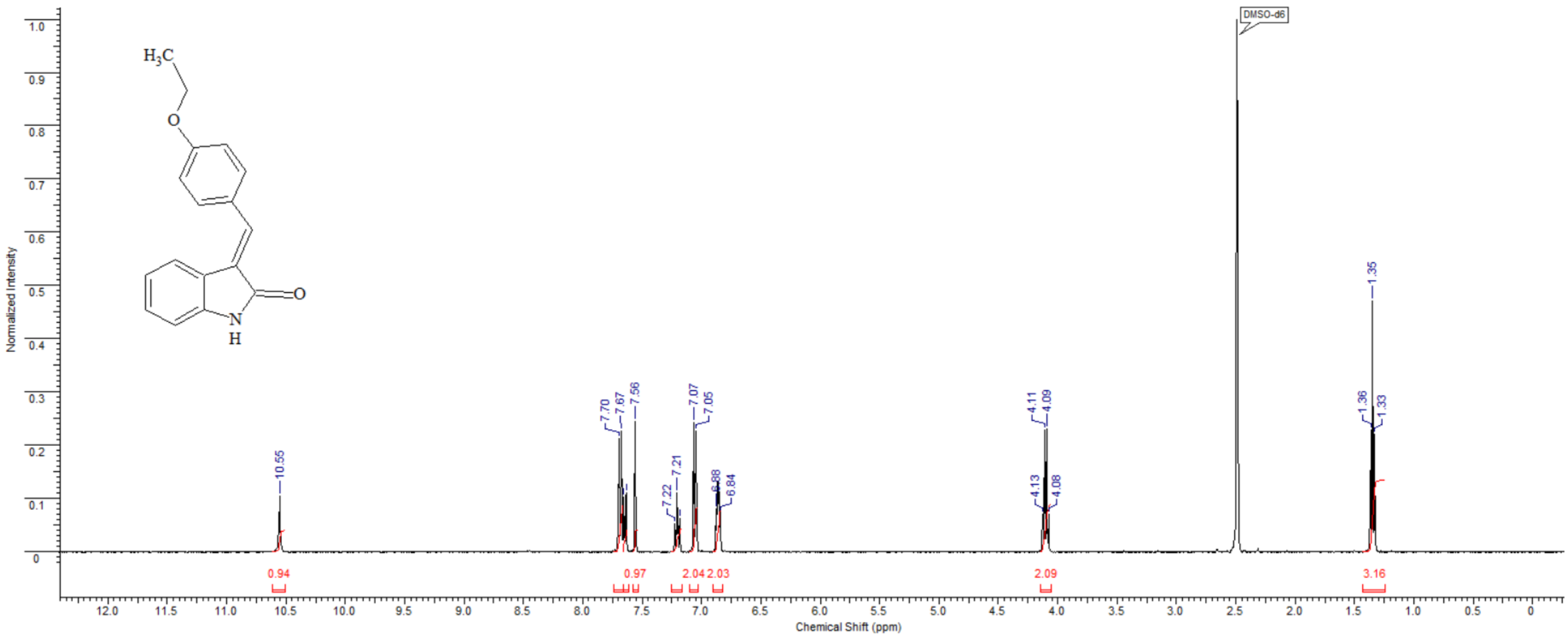

S10. 1H NMR spectrum of (*E*,*Z*)-3-(3,4,5-trimethoxybenzylidene)-2-oxindole **23**

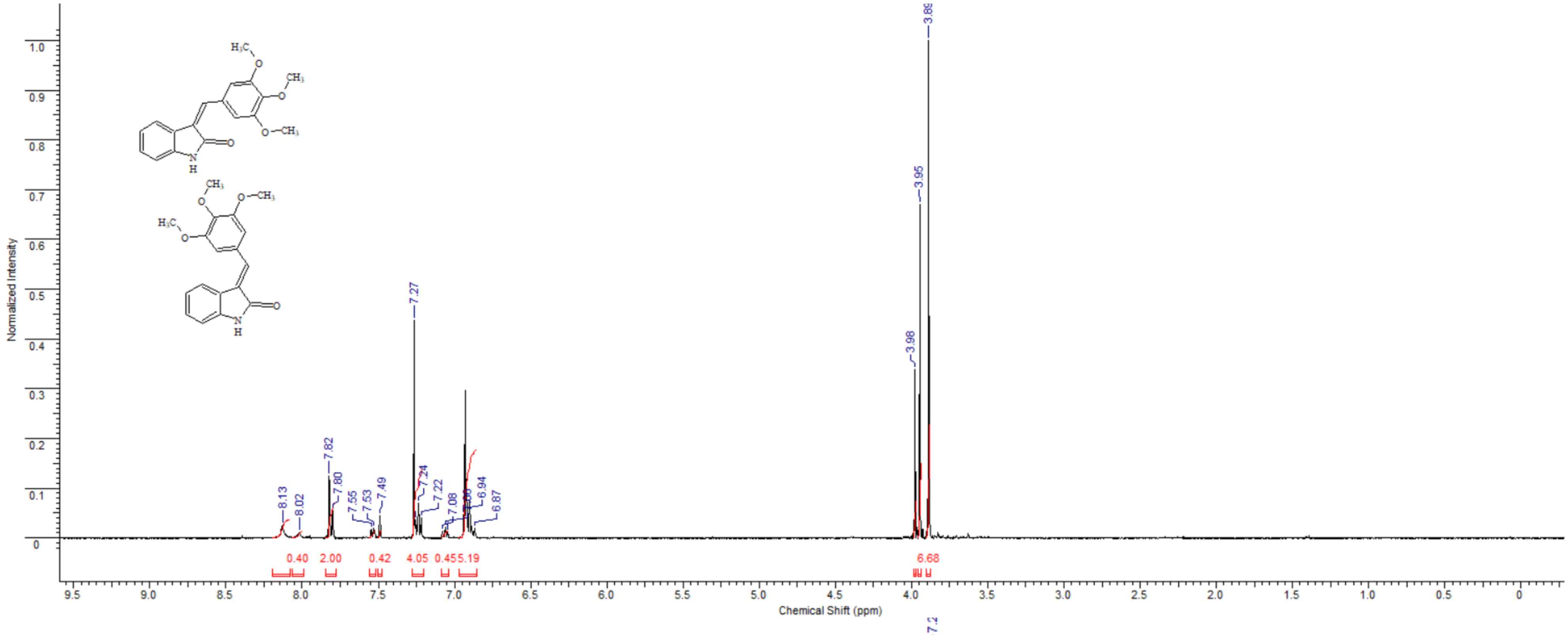


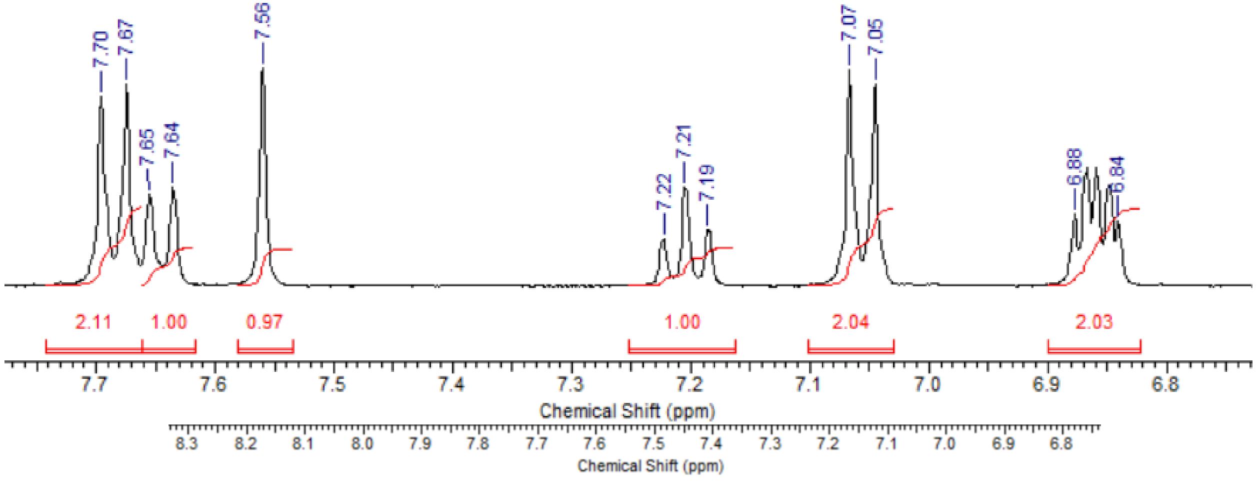

S11. 1H NMR (*E,Z*)-3-(3,5-dimethoxy-4-hydroxybenzylidene)-5-benzoylamino-2-oxindole **26**

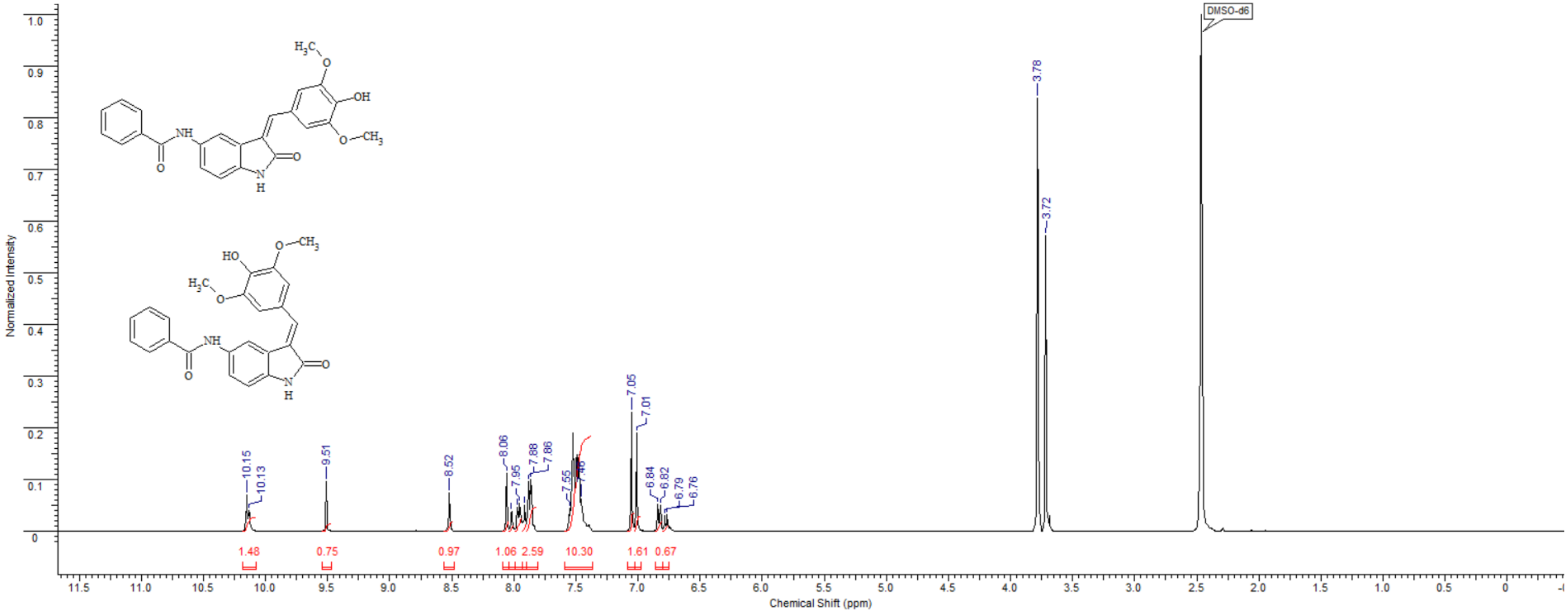

S12. 13C NMR spectrum of (*E*,*Z*)-3-(3,5-dimethoxy-4-hydroxybenzylidene)-5-benzoylamino-2-oxindole **26**

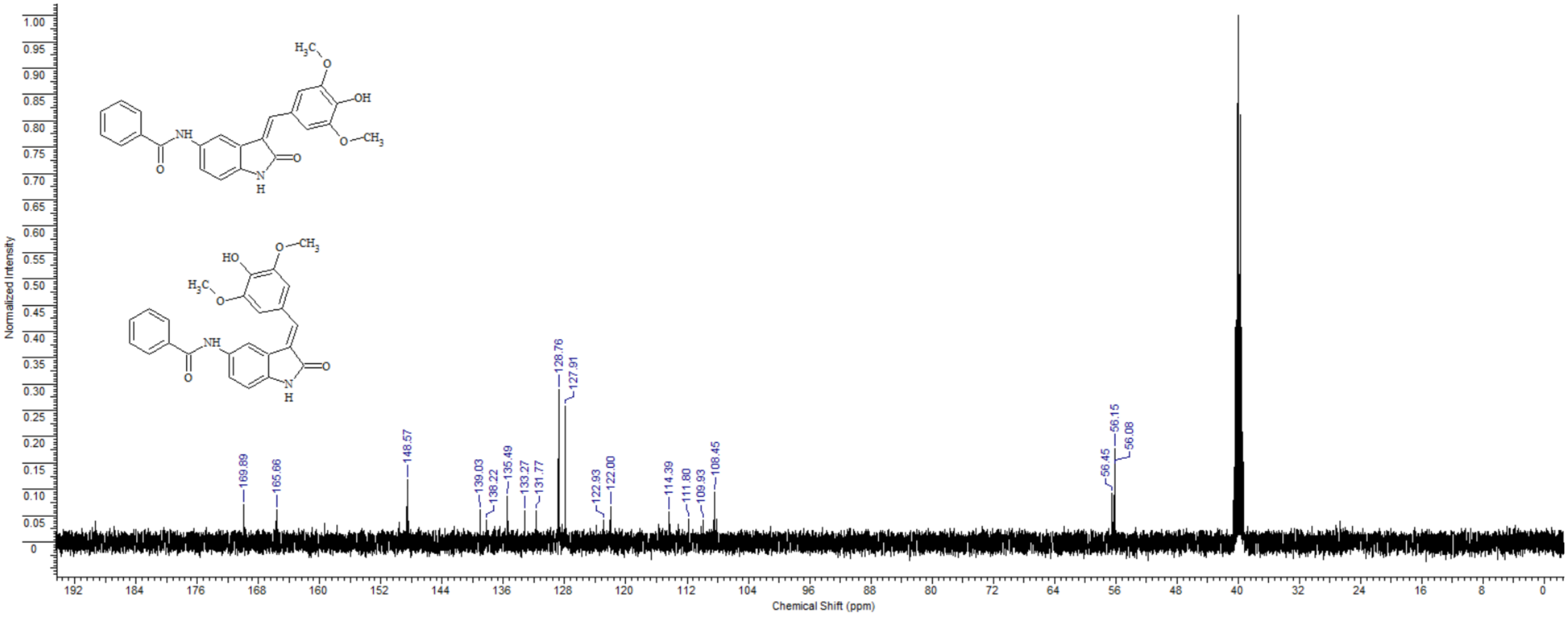

S13. 1H NMR spectrum of (*E*,*Z*)-3-(4-dimethylaminobenzylidene)-2-oxindole **30**

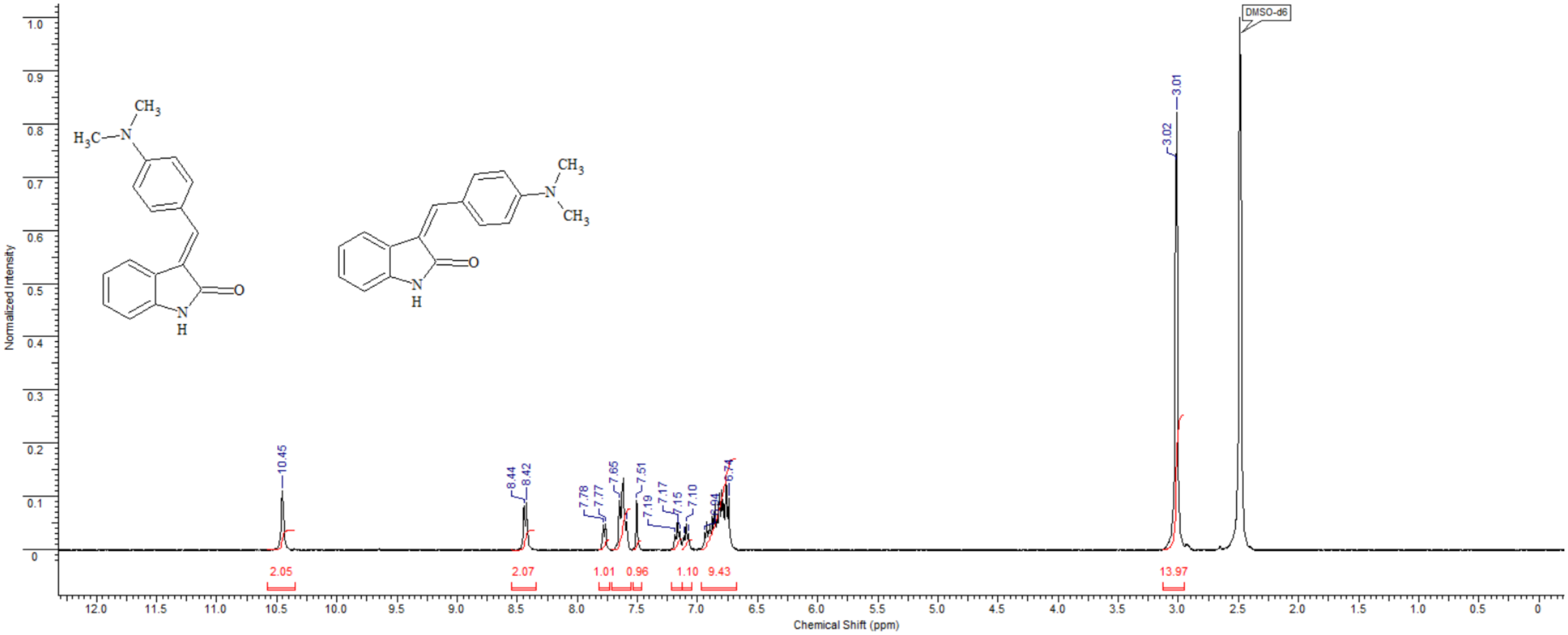

S14. 1H NMR spectrum of (*E*,*Z*)-3-(4-fluorobenzylidene)-2-oxindole **33**

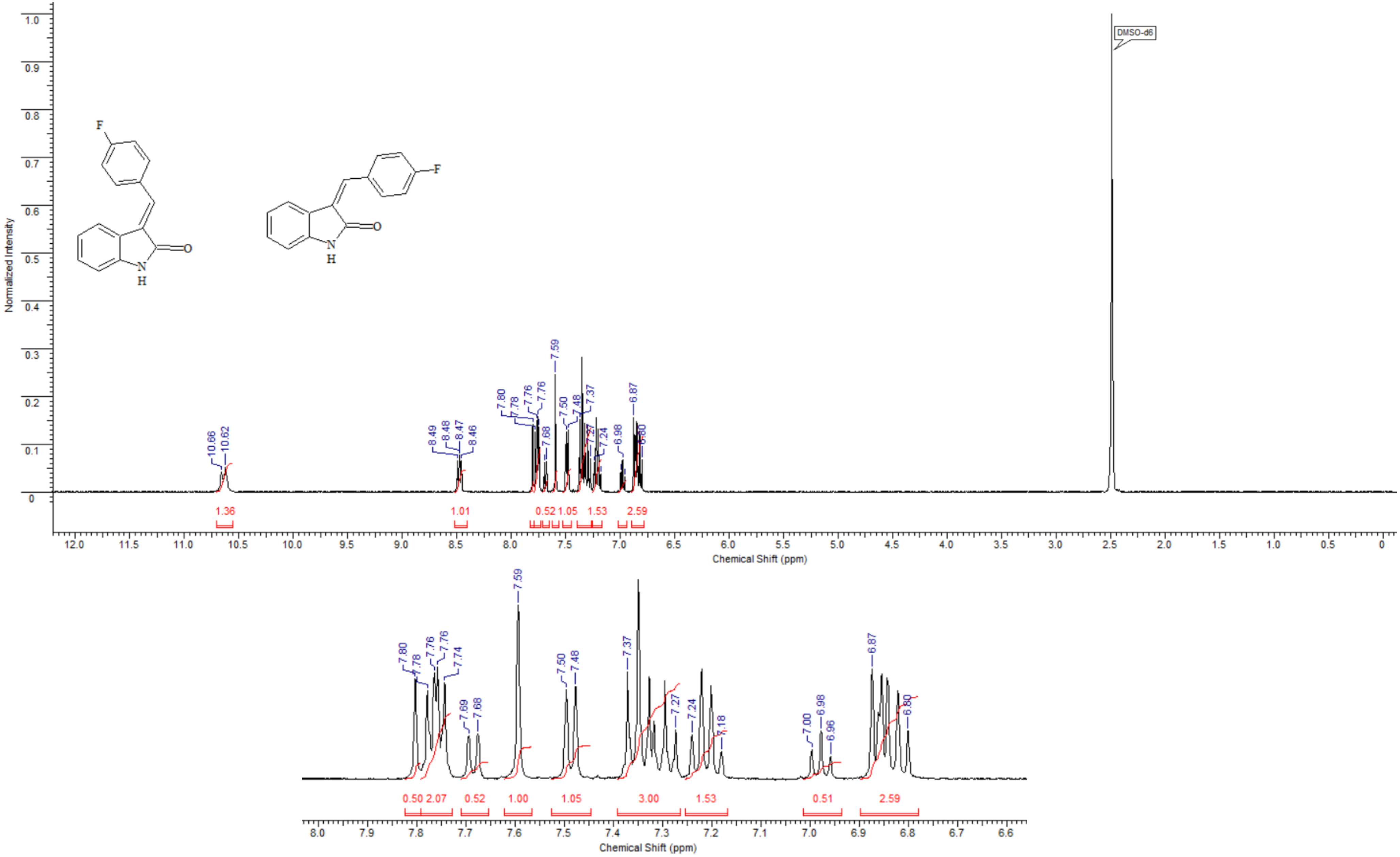

S15. 1H NMR spectrum of (*E*,*Z*)-3-(1-[2-(methoxycarbonyl)ethyl]-*1H*-pyrazol-4-ylmethylidene)-2-oxindole **45**

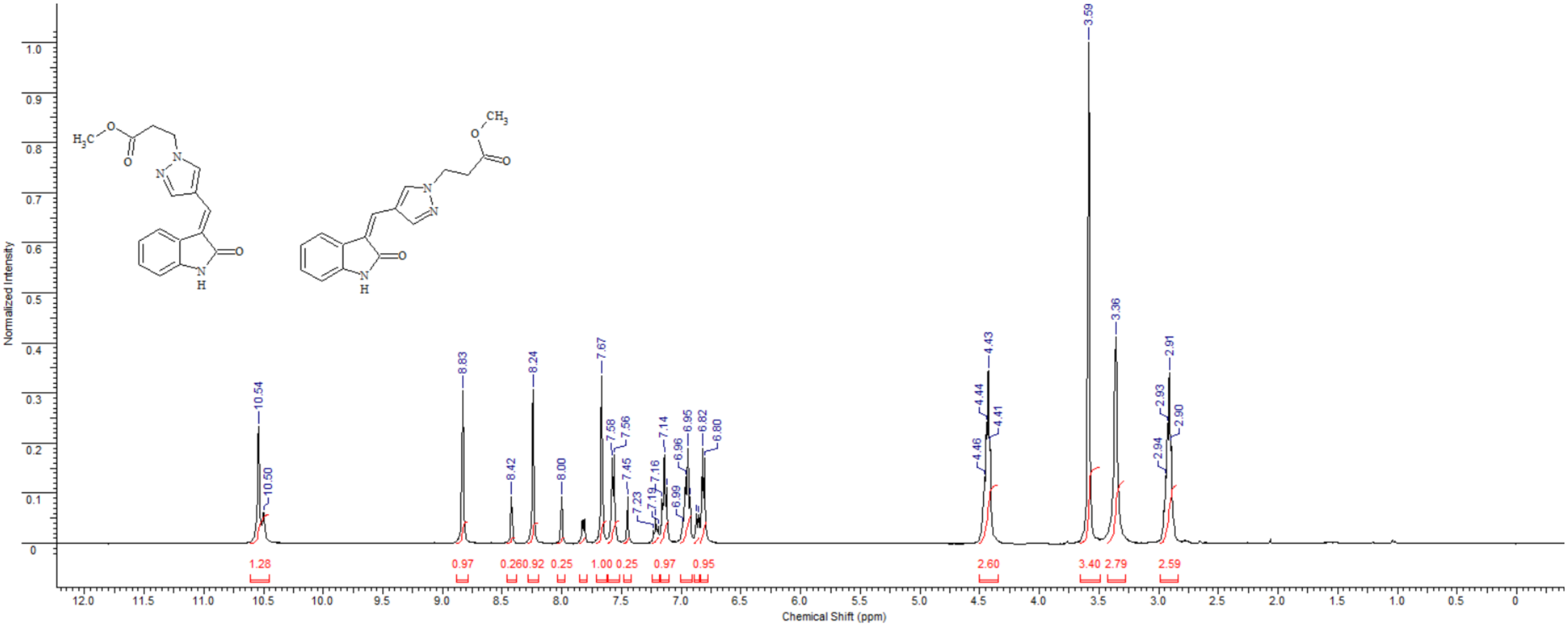

S16. 13C NMR spectrum of (*E,Z*)-3-(1-[2-(methoxycarbonyl)ethyl]-*1H*-pyrazol-4-ylmethylidene)-2-oxindole **45**

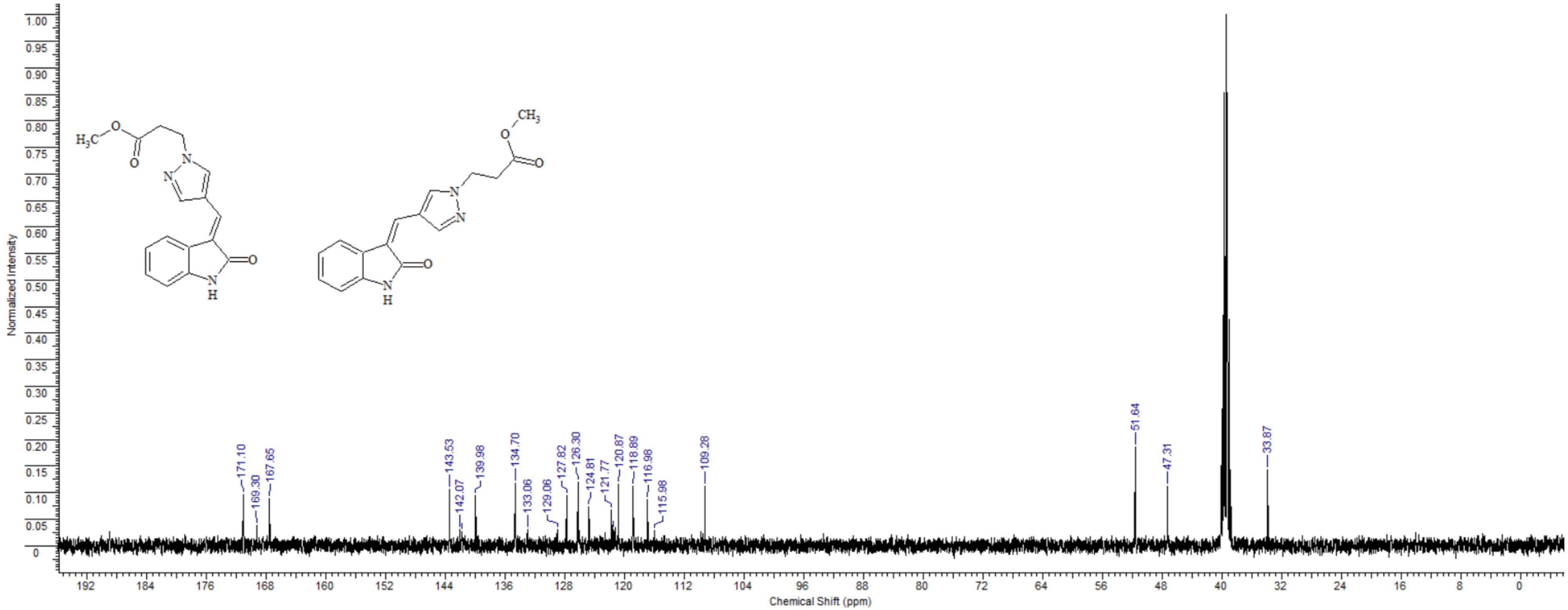

S17. 1H NMR spectrum of 3-(hydroxy(pyridin-2-yl)methyl)-2-oxindole **1a**

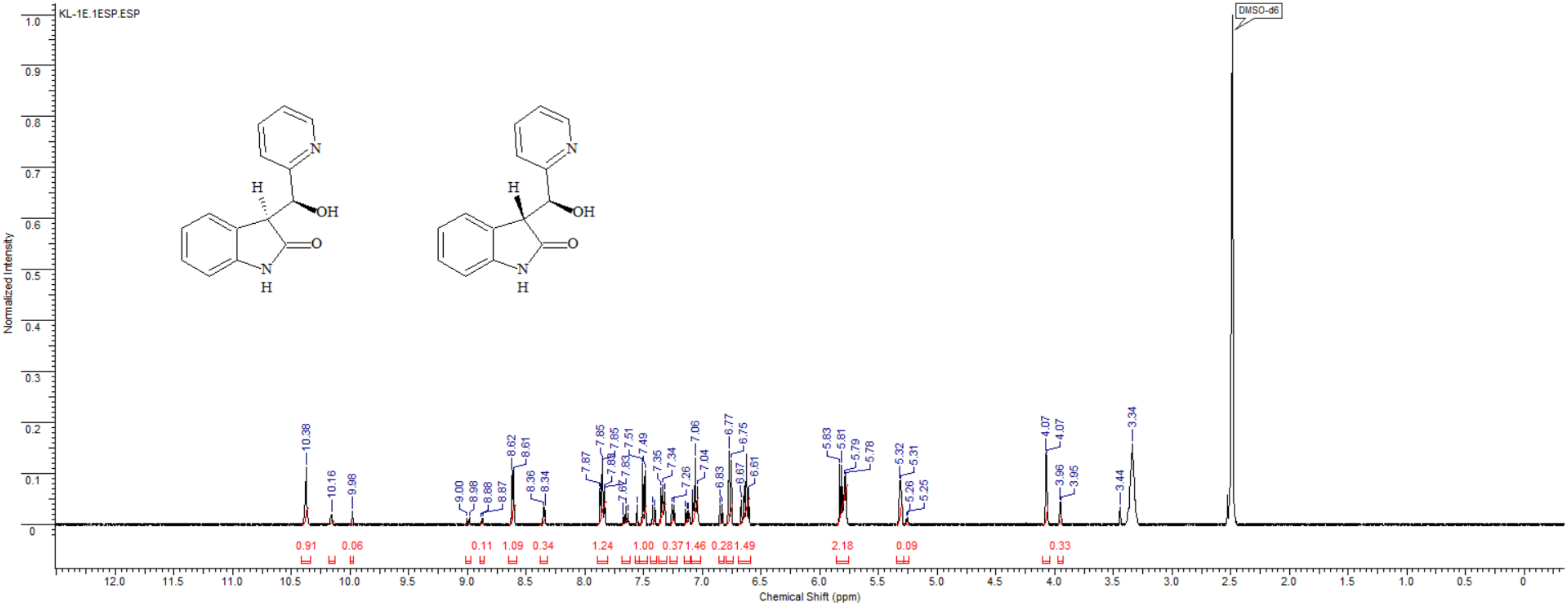

S18. 13C NMR spectrum of 3-(hydroxy(pyridin-2-yl)methyl)-2-oxindole **1a**

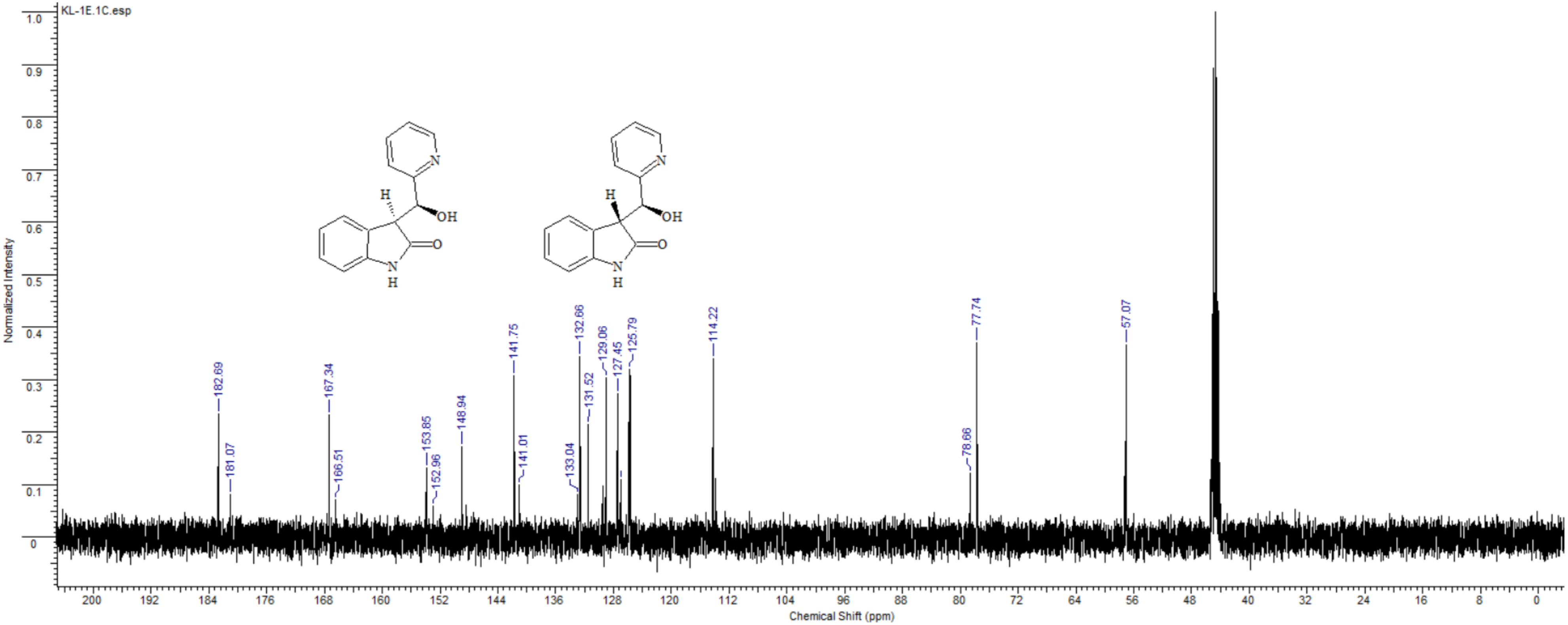

S19. 1H NMR spectrum of 3-pyridin-2-ylmethyl-5-amino-2-oxindole **47**

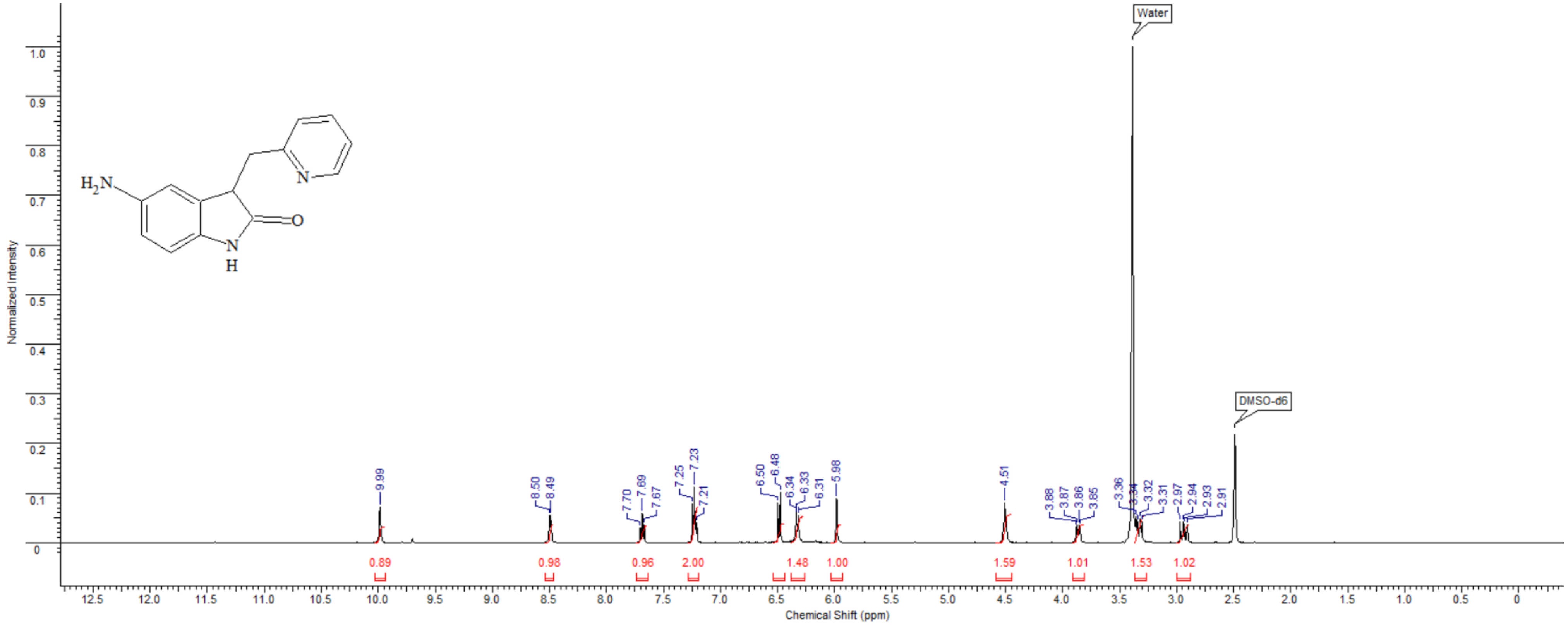

S20. 13C NMR spectrum of 3-pyridin-2-ylmethyl-5-amino-2-oxindole **47**

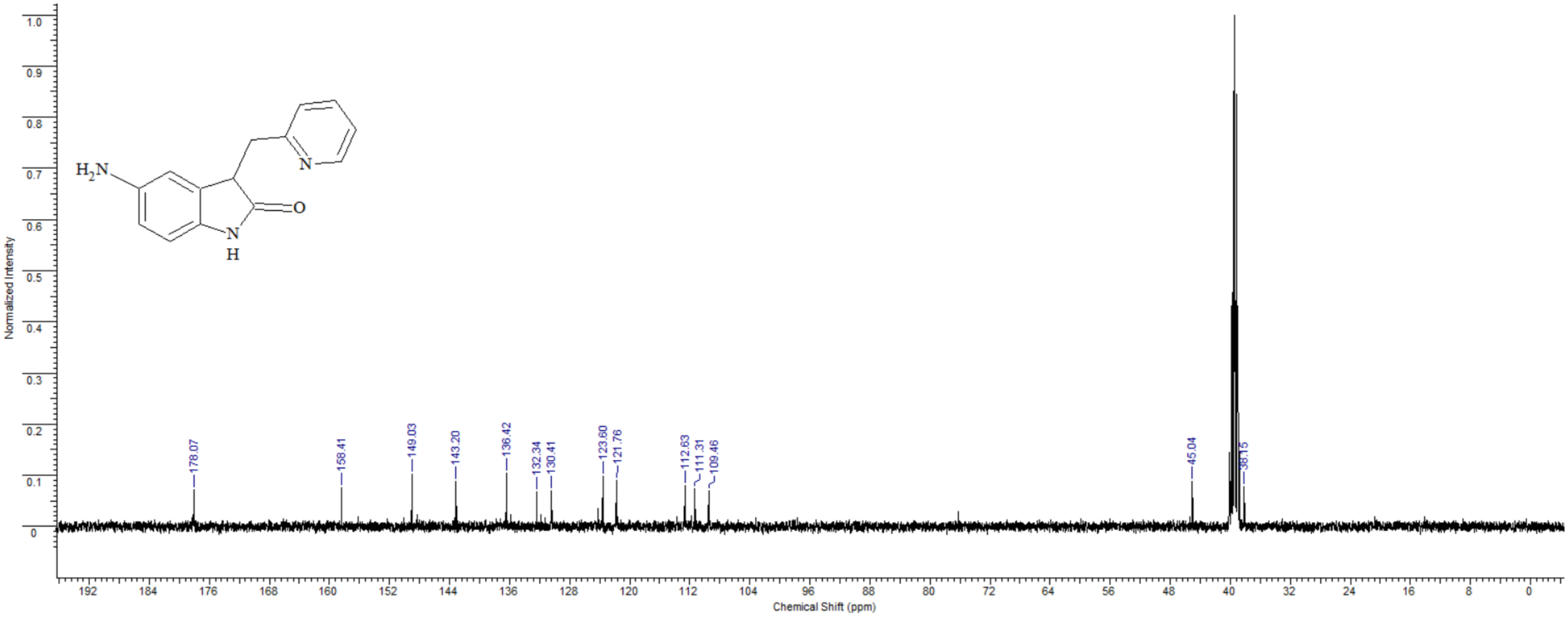

Biological data

S21. Concentration dependence of NQO2 inhibition by some active compounds

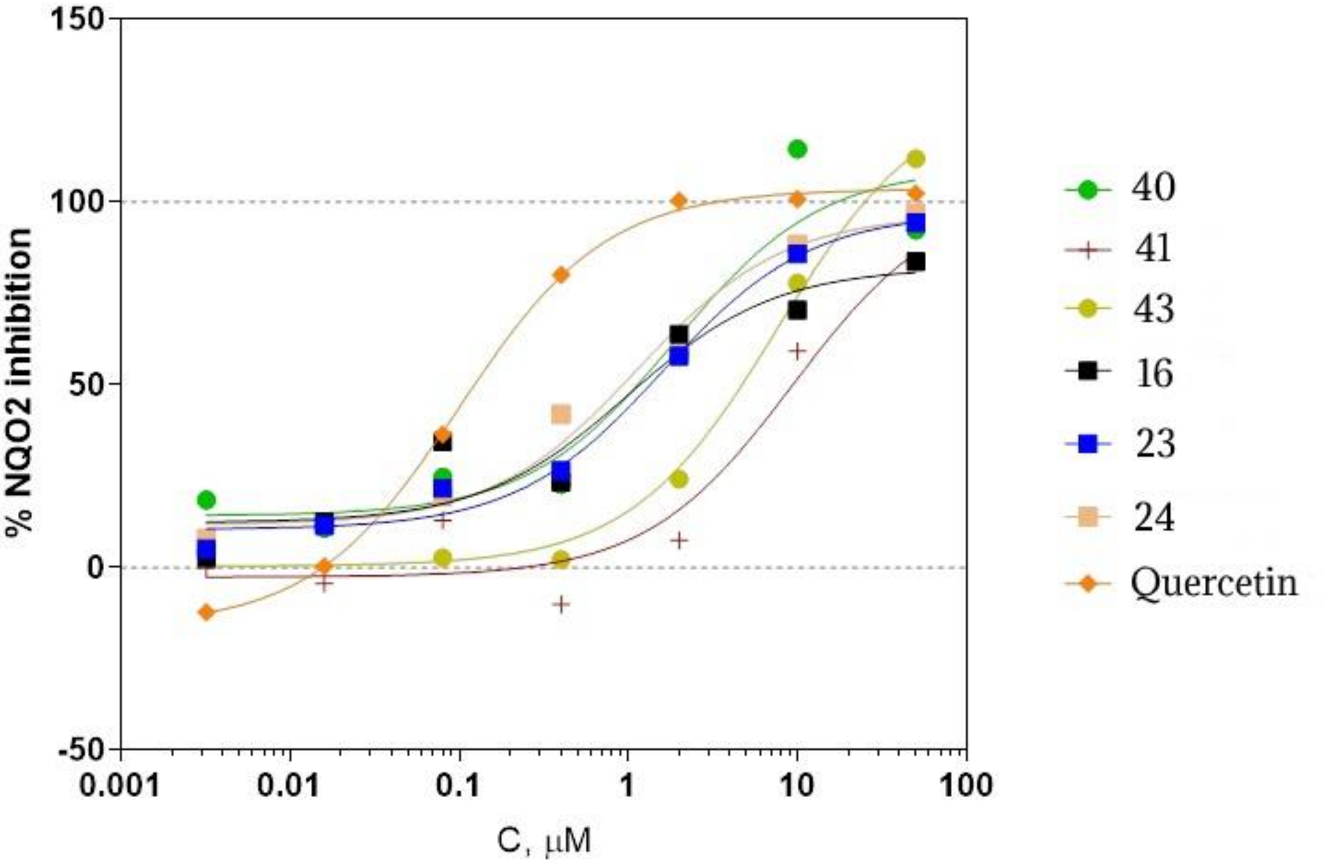


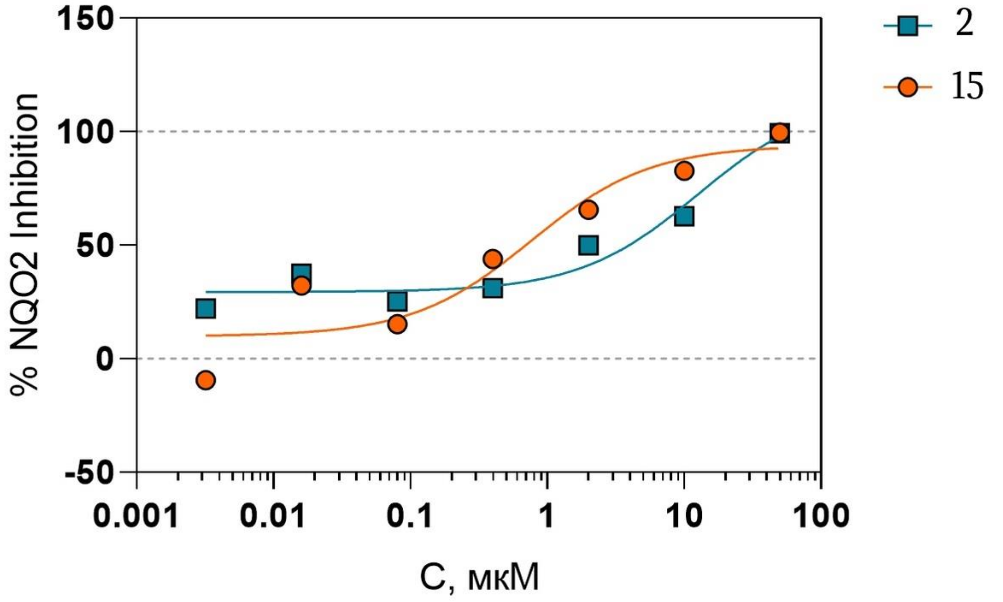

S22. Michaelis-Menten kinetic study for compound **15**

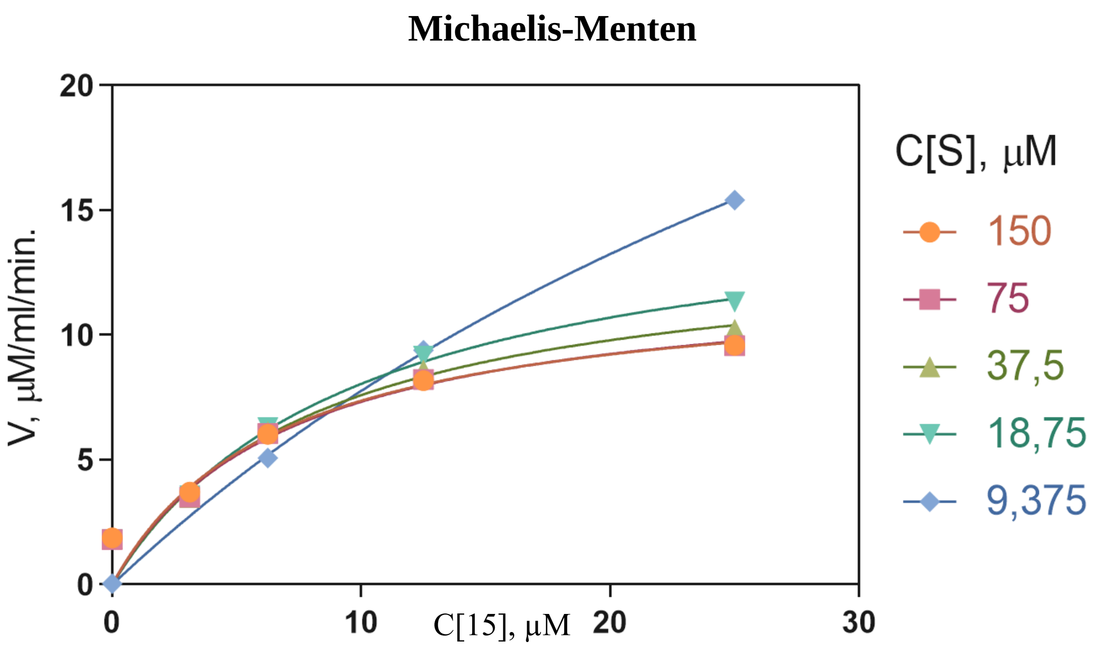

# Molecular modeling

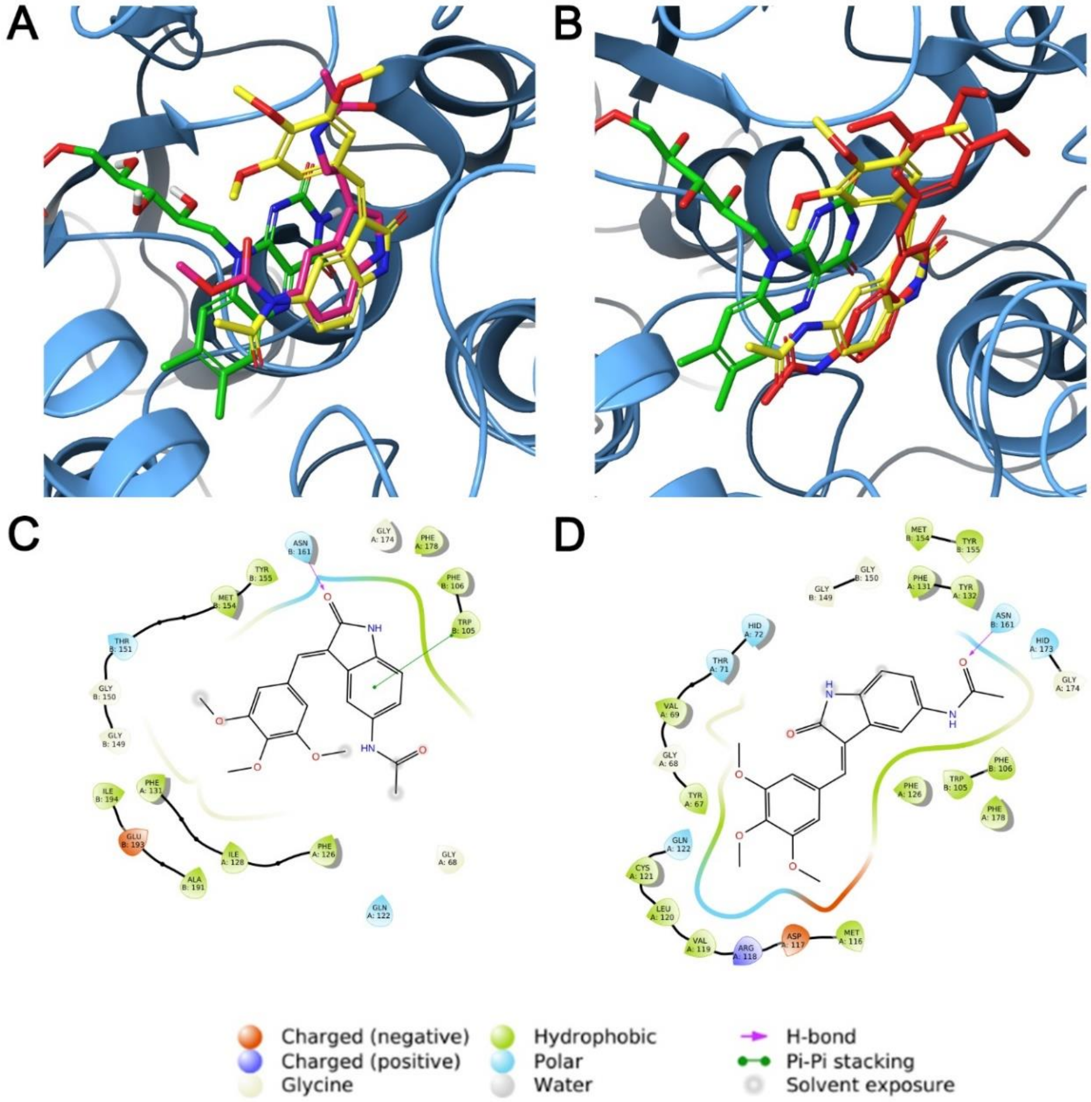


**Figure S23**. Proposed binding models of MCA-NAT and **24** to active site of NQO2 (blue) with FAD (green). **A**: binding pose of MCA-NAT (purple) and E (yellow) isomer of 24. **B**: the first pose of E (yellow) and the third one of Z (red) of **24**. **C**: interaction map for the E isomer of **24**. **D**: interaction map for the Z isomer of **24**.

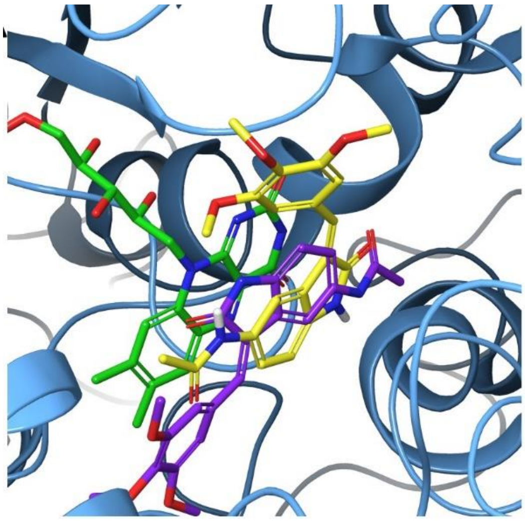

**Figure S24** the first pose of E (yellow) and the first one of Z (red) of **24**

**Table S1.** Docking score for E and Z isomers of selected compounds

| Compound | Docking score, kcal/mol | |
|---|---|---|
| | *E*-isomer | *Z*-isomer |
| **15** | -10.547 | -9.991 |
| **17** | -8.937 | -8.974 |
| **18** | -9.891 | -13.125 |
| **24** | -9.249 | -11.061 |
| **39** | -9.118 | -10.088 |
